# Inhalation of Low Molecular Weight Heparins as Prophylaxis against SARS-CoV-2

**DOI:** 10.1128/mbio.02558-22

**Published:** 2022-11-03

**Authors:** Julia Eder, Marta Bermejo-Jambrina, Killian E. Vlaming, Tanja M. Kaptein, Viktoria Zaderer, E. Marleen Kemper, Doris Wilflingseder, Sietze Reitsma, Godelieve J. de Bree, Danny M. Cohn, Teunis B. H. Geijtenbeek

**Affiliations:** a Department of Experimental Immunology, Amsterdam UMC, University of Amsterdam, Amsterdam, The Netherlands; b Amsterdam Institute for Infection and Immunity, Amsterdam, The Netherlands; c Institute of Hygiene and Medical Microbiology, Medical University of Innsbruck, Innsbruck, Austria; d Department of Pharmacy and Clinical Pharmacology, Amsterdam UMC, University of Amsterdam, Amsterdam, The Netherlands; e Department of Otorhinolaryngology, Amsterdam UMC, University of Amsterdam, Amsterdam, The Netherlands; f Department of Internal Medicine, Amsterdam UMC, University of Amsterdam, Amsterdam, The Netherlands; g Department of Vascular Medicine, Amsterdam UMC, University of Amsterdam, Amsterdam, The Netherlands; h Amsterdam Institute of Cardiovascular Sciences, Amsterdam, The Netherlands; University of Hong Kong

**Keywords:** low molecular weight heparin, SARS-CoV-2, infection prevention, virus-host interactions

## Abstract

New SARS-CoV-2 variants of concern and waning immunity demonstrate the need for a quick and simple prophylactic agent to prevent infection. Low molecular weight heparins (LMWH) are potent inhibitors of SARS-CoV-2 binding and infection in vitro. The airways are a major route for infection and therefore inhaled LMWH could be a prophylactic treatment against SARS-CoV-2. We investigated the efficacy of in vivo inhalation of LMWH in humans to prevent SARS-CoV-2 attachment to nasal epithelial cells in a single-center, open-label intervention study. Volunteers received enoxaparin in the right and a placebo (NaCl 0.9%) in the left nostril using a nebulizer. After application, nasal epithelial cells were retrieved with a brush for *ex-vivo* exposure to either SARS-CoV-2 pseudovirus or an authentic SARS-CoV-2 isolate and virus attachment as determined. LMWH inhalation significantly reduced attachment of SARS-CoV-2 pseudovirus as well as authentic SARS-CoV-2 to human nasal cells. Moreover, *in vivo* inhalation was as efficient as *in vitro* LMWH application. Cell phenotyping revealed no differences between placebo and treatment groups and no adverse events were observed in the study participants. Our data strongly suggested that inhalation of LMWH was effective to prevent SARS-CoV-2 attachment and subsequent infection. LMWH is ubiquitously available, affordable, and easy to apply, making them suitable candidates for prophylactic treatment against SARS-CoV-2.

## INTRODUCTION

Severe acute respiratory syndrome coronavirus 2 (SARS-CoV-2) emerged in Wuhan (China) in 2019 and quickly spread to the rest of the world, resulting in a global health crisis (1–3). SARS-CoV-2 belongs to the beta coronaviruses and causes coronavirus disease 2019 (COVID-19), an influenza-like disease ranging from mild respiratory symptoms to progressive inflammatory viral pneumonia, multiorgan disease, and death (4–6). Since 2020, the large-scale deployment of more than 30 approved vaccines has curbed viral spread and offered strong protection against severe disease and hospitalization (7–9). However, the continuous emergence of variants of concern (VoC), which are more contagious and potentially less susceptible to current vaccines, underscores the need for additional preventive methods and novel treatments for severe COVID-19 (10–13). It is also becoming clear that vaccinations are less effective in immunocompromised people (14–16). Thus, there is an urgent need for prophylactic treatments that prevent SARS-CoV-2 infections to address emerging VoC or protect vulnerable patient groups.

One of the routes of viral transmission is person-to-person via aerosolized droplets from the upper, conducting, and lower airways of infected people (17–19). Upon inhalation, droplets depending on the size can reach the upper or lower airways where infection can occur in airway epithelial cells (20). Therefore, a potential strategy for a prophylactic agent is to interfere with viral entry into airway epithelial cells, blocking infection at the earliest stage.

Angiotensin-converting enzyme 2 (ACE-2) is the main receptor used by SARS-CoV-2. The viral Spike (S) protein interacts with ACE-2 leading to viral entry into human cells (21–23). ACE-2 is expressed by different epithelial cells of the respiratory tract as well as alveolar macrophages (24, 25). Recent studies have shown that SARS-CoV-2 interacts strongly with heparan sulfate proteoglycans (HSPG) to attach to the cells, a prerequisite for infection via ACE-2 (26–28). HSPG are highly sulfated, negatively charged transmembrane receptors that are broadly expressed by different cells, including respiratory epithelial cells (29–31). In the nose, the olfactory neuroepithelium is rich in HSPG expression (32). HSPGs are also ubiquitously present in the lungs, where they are involved in maintaining the endothelial surface layer, pulmonary development, cellular signaling, and early immune activation (33, 34). A range of viruses, including HIV-1, hepatitis C virus (HCV), and human coronavirus NL63 exploit HSPG for cellular attachment (35–37). On epithelial cells, the HSPG family members Syndecan 1 and 4 have been shown to mediate SARS-CoV-2 attachment and infection (28). Heparin and low molecular weight heparins (LMWH) competitively block SARS-CoV-2 binding to epithelial cells (27, 38) and, thereby, prevent infection. LMWH has long been used in the clinic as anticoagulant therapeutics (39, 40), and LMWH might be used as a prophylactic treatment to prevent infection of SARS-CoV-2.

Here, we investigated whether *in vivo* nasal inhalation of LMWH enoxaparin protects against SARS-CoV-2.

## RESULTS

### Baseline characteristics.

**FIG 1:**
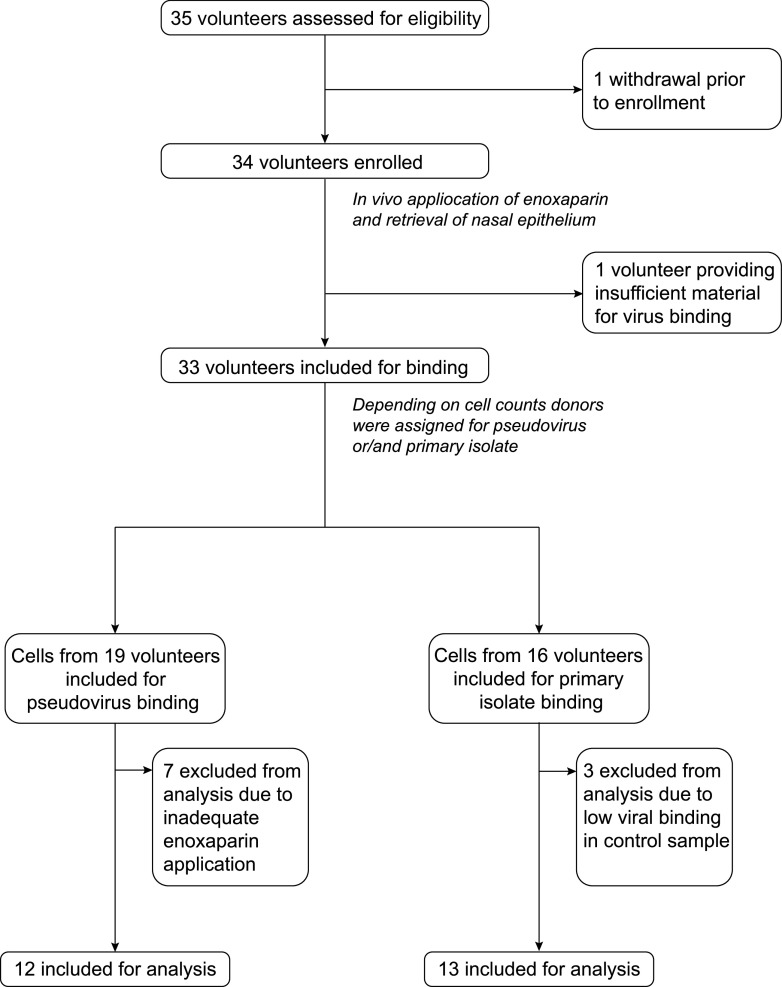
Flowchart of the volunteer inclusion. Volunteers were recruited within the AMC between January 7, 2021 and December 14, 2021. Both genders were eligible to participate. Inclusion criteria were an age between 18 and 65, good physical health (defined as not suffering from any illness or disease obstructing general daily functioning), and sufficient understanding of the Dutch language to comply to study procedures. Exclusion criteria were a positive SARS-CoV-2 antigen test (SARS-CoV-2 Antigen Rapid Test kit, JOYSBIO [Tianjin] Biotechnology Co. Ltd.), nasal-septum defects, usage of intranasal medication, frequent nosebleeds (>1/month), fever during the study visit (tympanic temperature >38.5°C during clinical visit), anamnestic or physical evidence of respiratory infection in the 4 weeks before the clinical visit, a known allergy or intolerance to LMWH or heparin-related products, a medical history of heparin-induced thrombocytopenia (HIT), the presence of mental disorders that would interfere with adherence to study procedures. Description of the inclusion process and parameters analysis, where 35 volunteers were assessed for eligibility, 1 volunteer withdrew before enrollment, and 34 volunteers were enrolled and received a placebo and 4500IE enoxaparin via nasal spray. Subsequently, the nasal epithelium was withdrawn and counted. One volunteer provided insufficient cellular material for all experiments and binding was subsequently not performed. Cells were plated and distributed into three categories for exposure to different viruses. A total of 19 donors provided material for pseudovirus binding. After the first 7 donors, we adapted the protocol due to the inefficient application of enoxaparin as measured by a tracer dye and these donors were excluded from the analysis. Next, 12 donors were included for analysis, 16 donors provided material for authentic SARS-CoV-2 virus binding, 3 donors were excluded from the analysis because the virus failed to bind in the placebo samples, and 13 donors were included for analysis. There was an overlap between donors, with 2 donors providing material for both SARS-CoV-2 pseudovirus and authentic virus binding.

A total of 35 volunteers were assessed for eligibility to participate in the clinical study (Fig. 1). One volunteer withdrew before enrollment, and 34 volunteers were enrolled. Vaccination status was assessed by way of self-reporting. Of the first seven volunteers, six were included before the start of the Dutch national SARS-CoV-2 vaccination campaign and were not vaccinated at inclusion. One volunteer was employed within health care and received one dose of the BioNTech/Pfizer mRNA vaccine as part of the early Dutch vaccination roll-out in January 2021. All other 27 volunteers were fully vaccinated according to national guidelines (1× COVID-19 vaccine Janssen, Janssen 2× Comirnaty, BioNTech/Pfizer, or 2× Spikevax, Moderna mRNA vaccine). After the initial 7 volunteers, we changed the procedure for the application of enoxaparin to provide a more homogenous coverage of enoxaparin. Therefore, all samples of these 7 volunteers were excluded from the analyses.

In total, 25 samples were included for virus binding, material for this was provided by 23 volunteers, with two volunteers providing samples for both viruses. Of these 13 were male and 10 female. Ages ranged between 18 and 65 years with the majority of volunteers being between 20 and 32 years of age with a median age of 33. Only two volunteers were above the age of 60. Details are displayed in Table 1.

**TABLE 1:** Baseline characteristics

Characteristic	All (*n* = 23)	SARS-CoV-2 pseudovirus (*n* = 12)	Authentic SARS-CoV-2 virus (*n* = 13)	Controls (*n* = 7) (only placebo)
Age^*a*^	33 (21–60)	33.2 (21–60)	31.4 (21–55)	31.9 (21–59)
Female	43.4%	33.3%	46.2%	42.9%
Vaccination status	100% fully vaccinated	100% fully vaccinated	100% fully vaccinated	14% partially vaccinated
SARS-CoV-2 infection in medical history	0%	0%	0%	0%
SARS-CoV-2 antigen test	100% negative	100% negative	100% negative	100% negative
Tympanic temp upon study visit^*a*^	36.7°C (36.0°C–37.4°C)	36.7°C (36.0°C–37.3°C)	36.7°C (36.1°C–37.4°C)	36.3°C (36.0°C–37.1°C)

a Age and tympanic temperature, mean (range).

### Enoxaparin prevented SARS-CoV-2 binding and infection of human epithelial cells *in vitro*.

**FIG 2:**
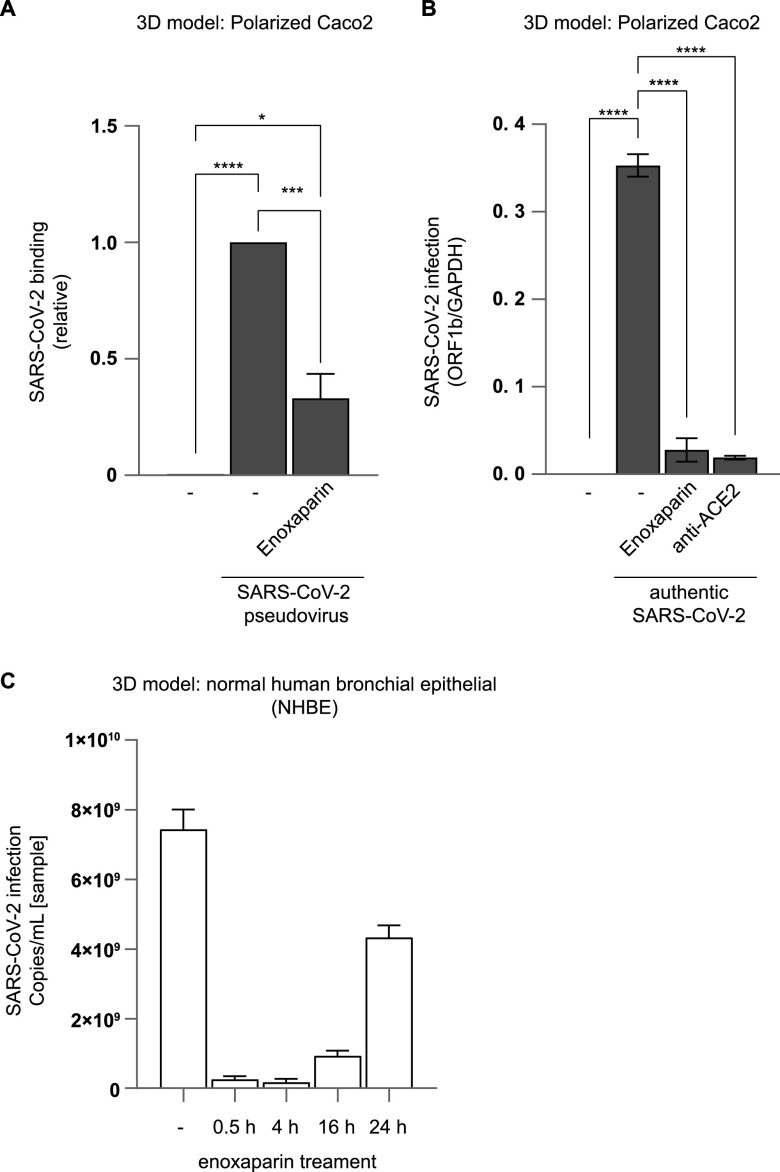
SARS-CoV-2 binding and infection of polarized epithelial cells was blocked by LMWH enoxaparin. (A) SARS-CoV-2 pseudovirus binding was measured in polarized Caco-2 cells that were cultured in a static 3D model. The virus was either directly added or upon prior incubation with an *in vitro* enoxaparin (250 IU) control for 30 min. Binding with SARS-CoV-2 pseudovirus was measured using a p24 ELISA. (B) Polarized Caco-2 were infected with authentic SARS-CoV-2 (Italy; 10^4^ TCID_50_) either without or in the presence of antibodies against ACE2 or after prior *in vitro* incubation with LMWH enoxaparin (250 IU) for 30 min. Authentic SARS-CoV-2 was detected after lysis by quantitative RT-PCR of viral RNA. (C) A 3D epithelial cell model of polarized normal human bronchial epithelial cells was treated with enoxaparin (nebulization) and SARS-CoV-2 was added at different time points (0.5 h, 4 h, 16 h, and 24 h). SARS-CoV-2 infection was measured by quantitative RT-PCR of viral RNA after 24 h. The data show the mean values and error bars are the SEM. Statistical analysis was performed using (A) one-way ANOVA with Tukey’s multiple-comparison test; *, *P* = 0.0203; ***, *P* = 0.0005; ****, *P* < 0.0001 (*n* = 2), or (B) one-way ANOVA with Tukey’s multiple-comparison test; ****, *P* < 0.0001 (*n* = 2). (C) *n* = 2.

SARS-CoV-2 pseudovirus strongly bound to polarized Caco-2 cells in a 3D epithelial model and binding was significantly inhibited by enoxaparin (*P* < 0.001) (Fig. 2A). The authentic SARS-CoV-2 isolate (wild-type [WT]) efficiently infected the polarized Caco-2 cells and infection was significantly inhibited by enoxaparin (*P* < 0.001) to a similar extent as observed with the blocking anti-ACE-2 antibody (Fig. 2B). These results suggested that SARS-CoV-2 pseudovirus and authentic SARS-CoV-2 bound to epithelial cells and enoxaparin effectively blocked SARS-CoV-2 binding and infection, supporting the use of enoxaparin as a prophylactic treatment against SARS-CoV-2 (28, 38).

To investigate how long enoxaparin remains effective after application, we performed a time course using a 3D epithelial cell model with primary normal human bronchial epithelial (NHBE) cells (41). NHBE cells were cultured for multiple weeks in transwells containing an air-liquid interphase (ALI), leading to the formation of intact ciliated pseudostratified epithelia with high mucus production, making this 3D respiratory model a suitable model for investigating the effect of drugs on epithelial tissues. Enoxaparin was sprayed from about 2.5 cm distance onto the apical side of fully differentiated epithelia to mimic enoxaparin distribution within the nasal cavity. To monitor how long the protective effect of enoxaparin against SARS-CoV-2 remained intact, NHBE cells were infected with authentic SARS-CoV-2 (WT) after enoxaparin exposure at different time points (0.5 h, 4 h, 16 h, and 24 h). Interestingly, enoxaparin protected the epithelial cells against infection for up to 16 h, and the protective effect was mostly lost at 24 h (Fig. 2C, Fig. S1C).

10.1128/mbio.02558-22.1 FIG S1 (A and B) Polarized normal human bronchial epithelial cells in the 3D epithelial cell model were exposed to enoxaparin (250 IU/mL) via nebulization and subsequently infected with authentic SARS-CoV-2 variants (MOI 0.1), WT, Delta (B.1.617.2) and Omicron (BA.5). Tissue integrity was monitored by TEER (Ω/cm^2^) measurements (A) using EVOM volt ohmmeter and SARS-CoV-2-RNA copy numbers/mL was determined (B). (C) A 3D epithelial cell model of polarized normal human bronchial epithelial cells was treated with enoxaparin (nebulization) and SARS-CoV-2 was added at different time points (0.5 h, 4 h, 16 h, and 24 h). SARS-CoV-2 infection was measured by quantitative RT-PCR of viral RNA after 24 h. Data show the mean values and error bars are the SEM. Statistical analysis was performed using (A) a two-way ANOVA with Tukey’s multiple-comparison test; ****, *P* < 0.0001 (*n* = 3); (B) a two-way ANOVA with Tukey’s multiple-comparison test; WT, **, *P* = 0.0017; Delta, *, *P* = 0.0189; Omicron, *, *P* = 0.0461 (*n* = 3) (C) (*n* = 2). Download FIG S1, PDF file, 0.2 MB. Copyright © 2022 Eder et al. 2022 Eder et al. https://creativecommons.org/licenses/by/4.0/ This content is distributed under the terms of the Creative Commons Attribution 4.0 International license.

To investigate the effect of enoxaparin on the WT strain of SARS-CoV-2 and two of the most recent and contagious VoC, NHBE cells were pretreated with enoxaparin for 30 min before the addition of different authentic SARS-CoV-2 viral strains (WT, and VoC Delta [B.1.617.2], and VoC Omicron [BA.5]) and kept in culture for 3 days. At day 3 postinfection, polarized NHBE cells were analyzed for transepithelial electrical resistance (TEER) (Fig. S1A), as an indicator for the integrity status of the tissue. TEER values significantly dropped upon SARS-CoV-2 infection with all strains compared to the uninfected condition, whereas enoxaparin preincubation restored TEER to the same level as uninfected cells (Fig. S1A). These data suggested that enoxaparin protected tissue integrity upon SARS-CoV-2 exposure by limiting infection. At day 3 postinfection, SARS-CoV-2 infection of the different variants was analyzed by RT-PCR (Fig. S1B). SARS-CoV-2 WT and the two VoCs, Delta (B.1.617.2) and Omicron (BA.5), efficiently infected polarized epithelial cells in the 3D model albeit at different efficiencies (Fig. S1B). Notably, enoxaparin blocked the infection of the three different strains as efficiently, suggesting that enoxaparin inhibition was independent of the SARS-CoV-2 variant.

We next investigated the efficacy of enoxaparin to block the infection of VeroE6 cells, which were highly susceptible to SARS-CoV-2. We determined the IC_50_ value for enoxaparin of SARS-CoV-2 binding to Vero cells at 156 IU/mL (Fig. S2A) and selected 250 IU/mL for subsequent *in vitro* experiments. We investigated whether there was a difference in inhibition when enoxaparin was applied to the cells or the virus. Either VeroE6 cells or authentic SARS-CoV-2 were exposed to enoxaparin and infection was measured. Preincubation of either SARS-CoV-2 or VeroE6 cells with enoxaparin inhibited SARS-CoV-2 binding (Fig. S2B), suggesting that enoxaparin interfered with SARS-CoV-2 infection at the cellular and viral levels.

10.1128/mbio.02558-22.2 FIG S2 (A) VERO E6 cells were exposed to authentic SARS-CoV-2 pretreated with different concentrations of enoxaparin (23.4 IU/mL to 3000 IU/mL) for 4 h before the binding was determined by RT-PCR. (B) The enoxaparin treatment effect was determined by virus binding to VeroE6 cells after 4 h. Enoxaparin was added either to authentic SARS-CoV-2 or the VERO E6 cells for 30 min before virus inoculation. (C) Infection of VeroE6 cells with authentic SARS-CoV-2 pretreated with different concentrations of enoxaparin (23.4 IU/mL to 3000 IU/mL) was determined after 24 h was determined by RT-PCR. (D) Nasal epithelial cells isolated from the nostrils of volunteers were treated with either a placebo or LMWH enoxaparin and were exposed to SARS-CoV-2 pseudovirus. (E) Nasal epithelial cells retrieved from the nasal cavity of healthy volunteers after exposure to placebo (saline solution, left nostril) and LMWH enoxaparin (right nostril) were exposed to authentic SARS-CoV-2 (hCOV-19 Italy) for 4h at 4C compared to an uninfected control sample Data show the mean values and error bars are the SEM. (B) One-way ANOVA with Tukey’s multiple-comparison test (virus versus treated virus; *, *P* = 0.0366); (virus versus treated cells; *, *P* = 0.0140); (treated virus versus treated cells; *, *P* = 0.0102) (A and C) *n* = 3 in duplicates; (C) at 375IU/mL with *n* = 1 due to technical issues), (B) *n* = 3 in monopole. (D) Two-way ANOVA with Tukey’s multiple-comparison test; ****, *P* < 0.0001 (*n* = 12). (E) Two-way ANOVA with Tukey’s multiple-comparison test; **, *P* = 0.0036; *, *P* = 0.0315 (*n* = 12). Download FIG S2, PDF file, 0.2 MB. Copyright © 2022 Eder et al. 2022 Eder et al. https://creativecommons.org/licenses/by/4.0/ This content is distributed under the terms of the Creative Commons Attribution 4.0 International license.

We further compared the efficiency of enoxaparin to block binding and infection of SARS-CoV-2. Our data showed that there was a higher background when blocking binding compared to infection, which was almost completely blocked by enoxaparin (Fig. S2C). At lower concentrations, enoxaparin was less efficient at blocking infection than binding. Because we used high enoxaparin concentration, our data suggested that the inhibition of binding corresponds to a block in infection.

### Characterization of nasal epithelial cells.

**FIG 3:**
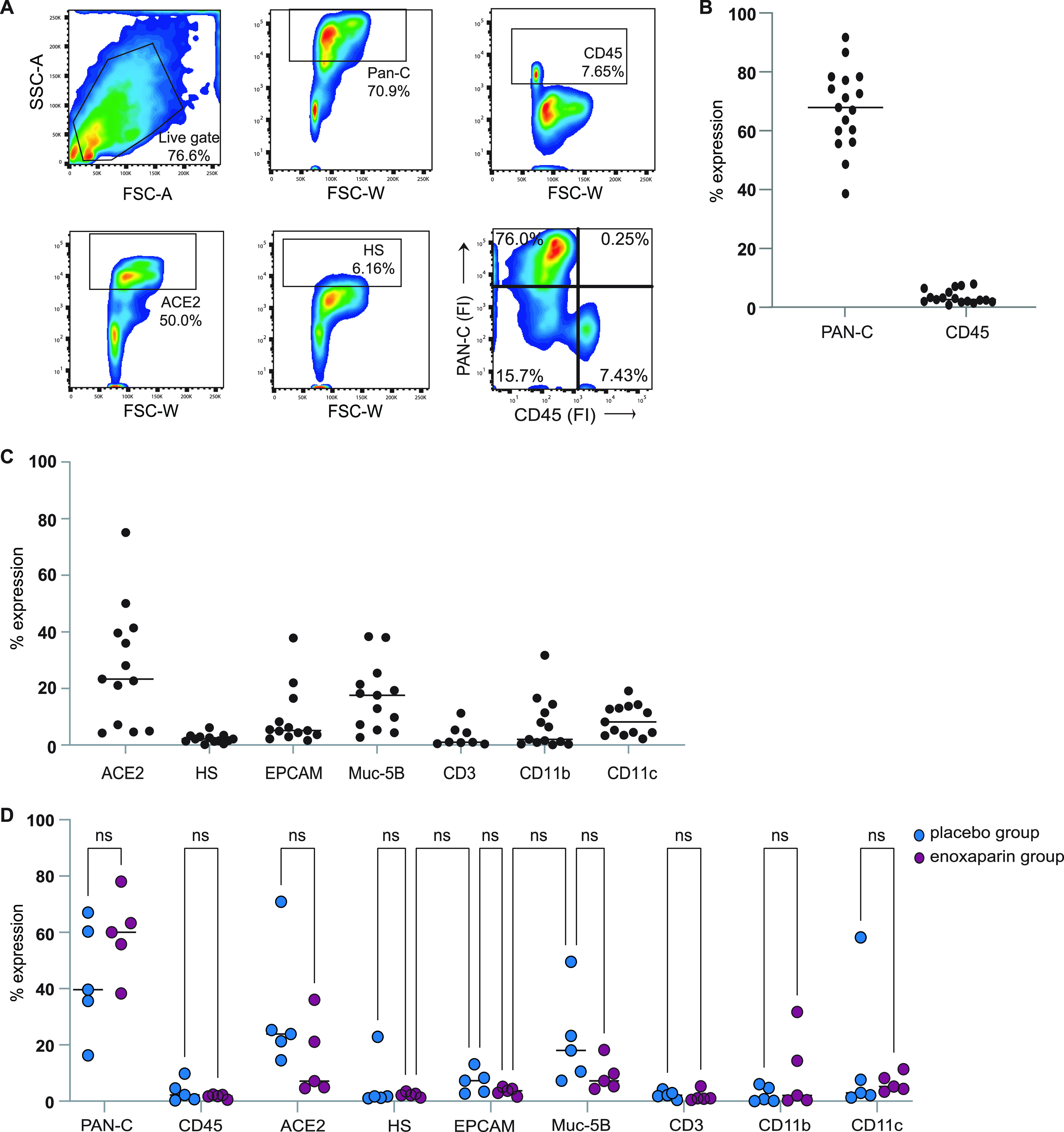
Characterization of primary human nasal epithelial cells. (A) Flow cytometry analysis of single-cell suspensions from the nasal epithelium. (A and B) Primary human nasal epithelial cells isolated from the nasal mucosa of healthy volunteers after treatment with either placebo (saline solution) or LMWH enoxaparin were directly labeled with pan-cytokeratin (Pan-C) and CD45. Additionally, cells were labeled with antibodies against ACE2 and heparan sulfate (HS) (*n* = 17). (C) Cells from the LMWH enoxaparin-treated nostril were additionally stained with antibodies against the surface markers EpCAM, Muc-5B, CD3, CD11b, and CD11c (*n* = 17, 4 donors excluded; CD3 *n* = 8). (D) Cells from both the placebo and LMWH enoxaparin-treated nose were stained for Pan-C, CD45, ACE2, HS, EpCAM, Muc-5B, CD3, CD11b and CD11c (*n* = 9, 4 donors excluded). The cellular phenotype was monitored using flow cytometry analysis. ns, not significant.

Volunteers inhaled both placebo and LMWH enoxaparin into separate nostrils, and we investigated the cellular fitness and composition of the isolated cells from each nostril. Nasal cells were stained epithelial and lymphocyte surface markers and analyzed by flow cytometry (Fig. 3A). The majority of cells expressed the epithelial marker pan-cytokeratin (42), whereas a small percentage of cells expressed CD45 (Fig. 3A and B), suggesting that the isolated cells were epithelial cells. Moreover, EpCAM and Mucin-5b were expressed by epithelial subsets. Importantly, cells isolated from the nasal cavity expressed ACE-2 and heparan sulfates (Fig. 3A and C), indicating their vulnerability to SARS-CoV-2 infection. Besides epithelial cells, a low percentage of lymphoid and myeloid cells, as indicated by expression of CD3, CD11b, and CD11c, were detected in the isolated cell fraction (Fig. 3C). Notably, no significant differences for the cell markers were detected between the placebo and enoxaparin treated cell samples (Fig. 3D), strongly suggesting that there was no interference of enoxaparin on cell receptor expression or cell activation upon treatment. These data suggested that nasal epithelial cells were a potential target for SARS-CoV-2 and that *in vivo* enoxaparin inhalation did not affect expression levels of SARS-CoV-2 receptors or cellular composition.

### Enoxaparin inhalation prevented SARS-CoV-2 pseudovirus from binding to nasal epithelial cells *in vivo*.

**FIG 4:**
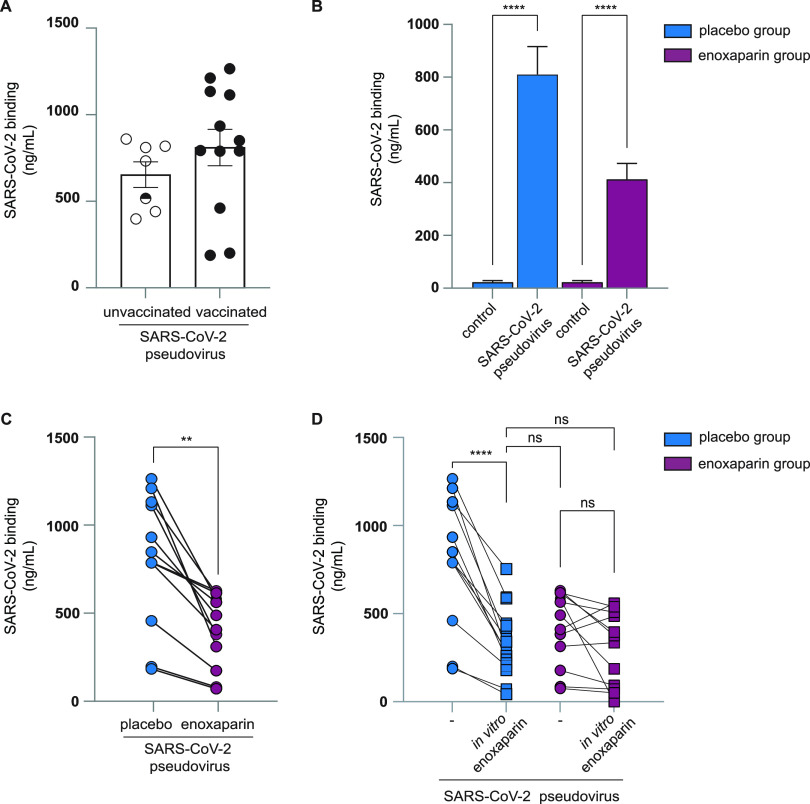
*In vivo* enoxaparin inhalation prevented SARS-CoV-2 pseudovirus from binding to nasal epithelial cells. (A) Nasal epithelial cells isolated from the placebo-treated volunteers (either unvaccinated [white circle, *n* = 6], partially vaccinated [white/black circle, n-1], or fully vaccinated [black circle, *n* = 12]) were exposed to SARS-CoV-2 pseudovirus. Binding was measured after 4 h by ELISA. (B and C) Nasal epithelial cells isolated from the nostrils of volunteers were treated with either a placebo or LMWH enoxaparin and were exposed to SARS-CoV-2 pseudovirus (D) in the presence or absence of an additional *in vitro* enoxaparin condition (250 IU) and binding was measured after 4 h by ELISA. Data show the mean values and error bars are the SEM. Statistical analysis was performed using (B) a two-way ANOVA with Tukey’s multiple-comparison test; ****, *P* < 0.0001 (*n* = 12), (C) a two-tailed, unpaired, nonparametric, Mann-Whitney test; **, *P* = 0.0043 (*n* = 12); or (D) a two-way ANOVA with Tukey’s multiple-comparison test; ****, *P* < 0.0001; ns, not significant (*n* = 12).

We isolated cells from the placebo-treated nasal cavity of volunteers that were either unvaccinated, partially vaccinated, or fully vaccinated at the time of treatment. The cells were subjected to SARS-CoV-2 pseudovirus for 4 h and binding was determined. Notably, no significant difference was observed between virus attachment to cells from unvaccinated and vaccinated individuals (Fig. 4A), suggesting that vaccination did not directly interfere with SARS-CoV-2 binding to nasal epithelial cells.

Next, we investigated the effect of *in vivo* enoxaparin treatment on *ex vivo* virus attachment. Cells isolated from placebo- and enoxaparin-treated volunteers were exposed to SARS-CoV-2 pseudovirus and the binding of SARS-CoV-2 was compared between both groups. SARS-CoV-2 pseudovirus bound efficiently to cells isolated from the placebo group (Fig. 4B and Fig. S2C). Strikingly, SARS-CoV-2 pseudovirus binding was significantly lower in the enoxaparin-treated group than the placebo group (Fig. 4B). Direct comparison of cells treated with LMWH enoxaparin or NaCl 0.9% from all donors supported the inhibitory effect of *in vivo* inhalation of enoxaparin on SARS-CoV-2 pseudovirus binding as nasal inhalation of enoxaparin significantly reduced of SARS-CoV-2 pseudovirus binding compared to the placebo group (*P* = 0.0003) (Fig. 4C). Moreover, we investigated whether the inhalation of enoxaparin was as efficient as *in vitro* preincubation with enoxaparin before virus exposure. We, therefore, compared the SARS-CoV-2 pseudovirus binding of placebo and enoxaparin group with binding when enoxaparin was added *in vitro* before virus exposure (Fig. 4D). The inhibitory effect of *in vivo* inhalation of enoxaparin was comparable to the *in vitro* addition of enoxaparin, suggesting that *in vivo* inhalation of enoxaparin was as efficient in inhibiting virus binding as can be achieved *in vitro*. These data suggested that *in vivo* inhalation of enoxaparin strongly inhibited SARS-CoV-2 pseudovirus binding to nasal epithelial cells.

### Enoxaparin inhalation blocked authentic SARS-CoV-2 attachment to nasal epithelial cells.

**FIG 5:**
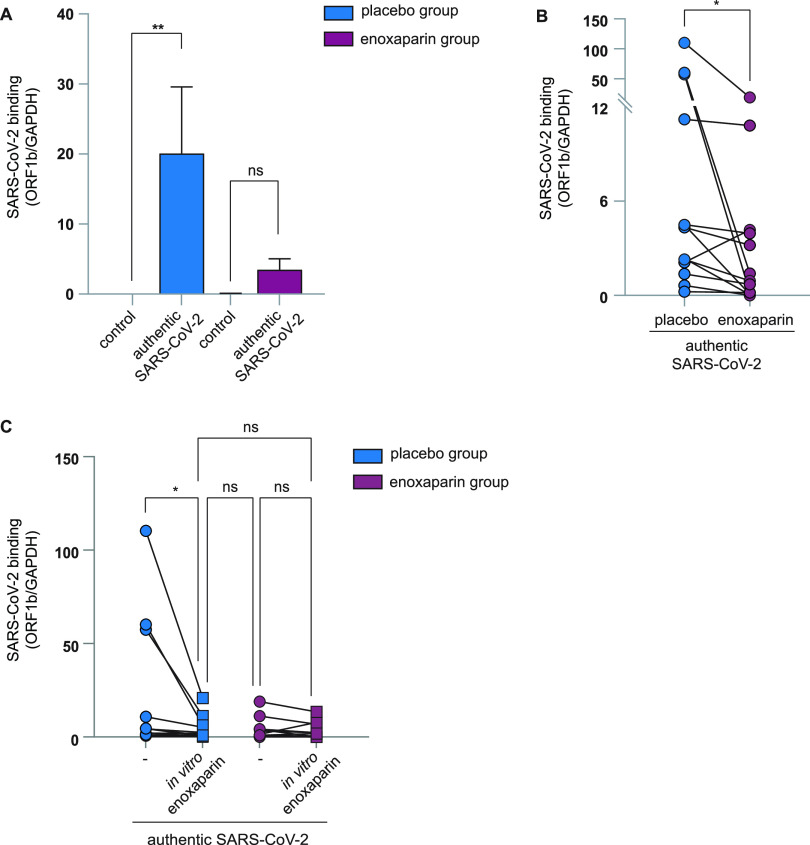
*In vivo* enoxaparin inhalation prevented authentic SARS-CoV-2 binding to nasal epithelial cells. (A and B) Nasal epithelial cells retrieved from the nasal cavity of healthy volunteers after exposure to placebo (saline solution, left nostril) and LMWH enoxaparin (right nostril) were exposed to authentic SARS-CoV-2 (hCOV-19 Italy) for 4 h at 4C compared to an uninfected control sample (A). (C) Additionally, authentic SARS-CoV-2 was incubated in the presence or absence of *in vitro* LMWH enoxaparin (250 IU) for 30 min before inoculation of epithelial cells. (A to C) Virus binding was measured after 4 h by real-time RT-PCR. Data show the mean values and error bars are the SEM. Statistical analysis was performed using (A) a two-way ANOVA with Tukey’s multiple-comparison test; **, *P* = 0.0036; *, *P* = 0.0315 (*n* = 12); or (B) a two-tailed, unpaired, nonparametric, Mann-Whitney test; **, *P* = 0.0387; ns, not significant.

We next compared the binding of an authentic SARS-CoV-2 isolate (hCOV-19/WT) to cells from placebo- and enoxaparin-treated volunteers. Freshly isolated cells were exposed to the authentic SARS-CoV-2 and binding was measured by quantitative RT-PCR. The authentic virus strongly bound to cells from the placebo group while the cells exposed to enoxaparin showed a decrease in virus binding (Fig. 5A and B, and Fig. S2E). We observed donor differences between the binding of SARS-CoV-2 to nasal cells but the binding of authentic SARS-CoV-2 isolate was significantly reduced (*P* = 0.049) when comparing the placebo and *in vivo* LMWH enoxaparin inhalation groups (Fig. 5A and B). Moreover, *in vivo* inhalation of enoxaparin was as efficient as *in vitro* preincubation of SARS-CoV-2 with enoxaparin as the observed inhibition of SARS-CoV-2 binding in the *in vivo* inhalation group was not further inhibited with *in vitro* preincubation of SARS-CoV-2 (Fig. 5C). Moreover, *in vitro* enoxaparin blocked SARS-CoV-2 in the placebo group as efficient as *in vivo* inhalation, strongly suggesting that *in vivo* inhalation of enoxaparin was as efficient in inhibiting SARS-CoV-2 binding to nasal cells as can be achieved *in vitro*. These data suggested that the protocol was suitable to investigate the effect of *in vivo* inhalation of enoxaparin or other reagents on SARS-CoV-2 binding.

### Adverse events and follow-up.

No adverse events (AE) or severe adverse events (SAE) occurred during the trial. A follow-up period of 24 h was maintained for all volunteers. This follow-up time corresponded with 5 times the half-life (t_1/2_) of enoxaparin. After 24 h follow-up, no adverse events were reported by volunteers.

## DISCUSSION

LMWH is commonly used as an anticoagulant and is considered safe, especially for external usage. Here, we performed a single-center, nonrandomized controlled trial where we investigated the potency of inhaled LMWH in preventing SARS-CoV-2 binding, which is a prerequisite to infection. Notably, *in vivo* inhalation blocked SARS-CoV-2 attachment to nasal epithelial cells from the human study participants. *In vivo* inhalation was as efficient as *in vitro* application, strongly suggesting that not only the developed protocol is suitable for investigating the protective effects of compounds against SARS-CoV-2 in an *in vivo* setting but also that inhalation is effective in blocking SARS-CoV-2 infection.

Although ACE-2 is the main receptor for SARS-CoV-2, several studies have shown that HSPG act as attachment receptors for SARS-CoV-2 and binding of SARS-CoV-2 to HSPG is required for infection (26, 28, 37). Unfractionated heparin or different LMWH interfere with SARS-CoV-2 binding to HSPG and, therefore, prevent infection *in vitro* (27, 38). Because *in vitro* infections depend on prior binding to HSPG, it is tempting to speculate that *in vivo* infection is even more dependent on first attachment to HSPG due to airflow and low virus concentrations. Interestingly, our data showed that preincubation of either SARS-CoV-2 or epithelial cells with LMWH blocked SARS-CoV-2 binding and infection, further supporting the use of heparins and LMWH to prevent SARS-CoV-2 infection and transmission (27, 28, 38).

In current clinical practice, heparins, and their LMWH counterparts, are given as an antithrombotic agents in pulmonary, cardiac, and vascular medicine (39, 43, 44). The use of these anticoagulants is widespread and considered safe (45). Studies involving inhaled heparins have shown that bronchially and pulmonary applied heparins do not increase plasma anti-Xa activity even in high doses (45–47). We analyzed the nasal cells isolated from the volunteers by flow cytometry and observed that the majority of cells expressed epithelial markers. Only low percentages of lymphoid and myeloid cells were detected. Interestingly, enoxaparin inhalation did not affect the cellular composition, and we did not observe an increased influx of lymphocytes. These data suggested that enoxaparin inhalation did not lead to inflammation. We also did not observe any adverse effects of the enoxaparin inhalation in the healthy volunteers. These data, therefore, suggested that nasal enoxaparin inhalation was safe and did not significantly change the mucosal integrity.

Elimination of enoxaparin from the body happens through direct filtration in the kidneys (10% of the total dose) or metabolic degradation via desulphurization and depolymerization in the liver to smaller fragments (48). While the half-life is well understood in humans, being approximately 5 h after a single subcutaneous dose (48), the longevity of LMWH in mucosal tissues is poorly understood. Interestingly, enoxaparin application to the 3D epithelial cell model of normal human bronchial epithelial cells protected against infection with authentic SARS-CoV-2 for up to 16 h, suggesting that enoxaparin remains longer effective in mucosal tissues compared to subcutaneous injection.

We observed variations in virus attachment between different volunteers, which could be caused by variations in susceptibility to SARS-CoV-2. We observed differences between ACE-2 expression but there was no correlation between the observed binding variations. Moreover, anatomical and physiological differences exist between volunteers, which may affect either the effect or the final dose applied to the mucosal surface. Interestingly, we did not observe any differences between SARS-CoV-2 pseudovirus binding to epithelial cells from vaccinated and unvaccinated volunteers, indicating that vaccination status did not cause the observed variations in virus attachment.

Our data confirmed that a vast majority of cells covering the nasal mucosa were of epithelial origin, and the presence of both ACE-2 and HSPG on the nasal epithelium in our study suggested these cells were at risk of infection by SARS-CoV-2 (24, 49). In the current study, we observed a strong binding of SARS-CoV-2 pseudovirus to the cells isolated from the nose, suggesting that indeed SARS-CoV-2 efficiently interacts with nasal epithelial cells and this might lead to infection of the host. Inhalation of enoxaparin effectively blocked the binding of SARS-CoV-2 pseudovirus as well as authentic SARS-CoV-2 to the nasal cell fraction.

These data demonstrated that enoxaparin inhalation prevented the interaction of SARS-CoV-2 to nasal cells and this might be used as prophylaxis. Moreover, it suggested that HSPGs were relevant in human susceptibility to SARS-CoV-2. Our study further showed that the application of enoxaparin *in vivo* is as efficient in blocking SARS-CoV-2 binding as *in vitro* addition of enoxaparin before virus binding (28, 38). These findings underscore the relevance of LMWH as inhalation prophylaxis to SARS-CoV-2 and showed that our current protocol was suitable to study interventions to prevent SARS-CoV-2 binding.

Inhibition of binding was taken as a measure of protection because several studies show that blocking the binding of SARS-CoV-2 prevents infection as binding is a prerequisite for ACE-2-dependent infection (28, 50–52). Indeed, enoxaparin blocked both binding and infection of SARS-CoV-2 in the 3D primary human epithelial model, with the protection, offered not only for the SARS-CoV-2 WT isolate but also in the Delta (B.1.617.2) and Omicron (BA.5) variants, suggesting that enoxaparin inhibition is insensitive to changes observed in current VoC and might be a broad inhibitor of SARS-CoV-2 and VoCs.

In the volunteer study, we focused on SARS-CoV-2 binding as a measure of infection as it is a short procedure allowing assessment of inhalation treatment. Infection of SARS-CoV-2 requires culturing of the primary isolated nasal cells at 37°C for 24 h, which would affect cell viability and more importantly differentiation state of the cells. By comparing SARS-CoV-2 binding to nasal cells isolated from the placebo and *in vivo* enoxaparin inhalation groups we were able to assess enoxaparin efficacy as prophylaxis without major confounding factors.

Because infection of humans with SARS-CoV-2 in a study setting poses several ethical challenges, especially because we cannot predict the severity of COVID-19 in healthy individuals, we designed a nonrandomized controlled trial involving *in vivo* medication and *ex vivo* virus exposure. While the first human SARS-CoV-2 challenge study was recently completed (53), safety concerns remain (54).

The risk of using heparin or LMWH in a preventive setting would be loss of hemostasis. We did not observe any bleeding or other adverse effects in the study participants. Previous research has investigated the bronchial application of heparins in the context of asthma and has shown that LMWH does not lead to an increase in bleeding. Studies conducted revealed that various heparins could be inhaled at doses higher than current therapeutic levels without an increase in bleedings or alteration of both prothrombin time and activated partial thromboplastin time when comparing study groups receiving nebulized heparins compared to best clinical practice (55, 56). The current clinical practice uses enoxaparin at a dose of 100 IU/kg. Ahmed et al. (55) nebulized either 200 IU/kg enoxaparin or 80,000 IU unfractionated heparin and found no adverse events in the trial population. We have demonstrated efficient inhibition of SARS-CoV-2 binding using a total of 4500 IU enoxaparin. *In vitro* addition of enoxaparin did not further decrease binding supporting the efficiency of *in vivo* block by inhalation. This indicated that inhalation of enoxaparin was a suitable way to protect the epithelium and the concentration used in this study resulted in a maximum block.

Interestingly, critically ill COVID-19 patients that were treated with LMWH, resulted lower mortality (57). An explanation is that this reduction in mortality is due to the anticoagulative properties of LMWH and their associated reduction in pulmonary thrombotic events (58, 59). Yet, there is persistent evidence that LMWH has an advantage over other anticoagulants as they also curtailed the duration of the SARS-CoV-2 infection (60). Additionally, administering LMWH to COVID-19 patients is associated with a shorter time to SARS-CoV-2 swab negativity in the case of infection (61). Similarly, it has been observed that competition of LMWH with HSGP to bind SARS-CoV-2 mitigates the chance of a cytokine storm (62). This might indicate a powerful method to prevent or curb outbreaks of SARS-CoV-2 VoC or novel future CoV epidemics.

With the rise of new VoC that contain mutations allowing them to evade immunity gained by vaccines or prior SARS-CoV-2 infection (63), it has become paramount to find alternative strategies to prevent SARS-CoV-2 infection and transmission. It is even possible that VoC has a higher affinity for HSPG as recently suggested (64), which supports the prophylactic use of enoxaparin. Mutations in the surface of the omicron VoC render the virus highly positively charged, suggesting a higher affinity to HS (65). Preliminary data with leftover nasal epithelial cells showed that the VoC Delta bound to nasal cells from the placebo group and was blocked by *in vivo* enoxaparin inhalation similar to the SARS-Cov-2 primary isolate (hCoV/WT), indicating that *in vivo* inhalation of enoxaparin is a prophylactic to prevent VoC infections. However, due to insufficient material, we were unable to power this accordingly. Nevertheless, a reduction in infection of both delta and omicron variants was observed in our 3D epithelial model, indicating a similar mechanism as with the authentic isolate.

To date, we and others have proposed the use of heparin or LMWH to prevent SARS-CoV-2 infection and transmission (27, 38). Our data suggested that direct *in vivo* application of LMWH enoxaparin to the nasal epithelium prevents SARS-CoV-2 attachment, a prerequisite for infection and transmission of different SARS-CoV-2 variants.

In conclusion, we demonstrated that LMWH inhalation was an efficient and safe prophylactic treatment preventing SARS-CoV-2 attachment to nasal epithelial cells. Our *in vivo* exposure/ex vivo analysis model allows us to mitigate the risk to human volunteers while preserving the ability to work in as close-to-real setting as possible. Furthermore, we severely limit the risks inflicted on our volunteers using ex vivo virus exposure. The use of brushes to retrieve nasal epithelial cells also reduces the burden on our volunteers. LMWH is relatively inexpensive and widely available, and previous experience with nasal/bronchial application of these medications reveals it to be safe. This could make LMWH an excellent tool to limit the spread of SARS-CoV-2 and possible new VoC.

## MATERIALS AND METHODS

### Study design.

We designed a single-center, nonrandomized controlled trial conducted at the Amsterdam UMC in Amsterdam. The study was registered at the European drug regulatory affairs for Clinical Trials (EudraCT) under code 2020-003992-16, at the Dutch clinical trial registry under code NL9430, and was conducted following the principles of Good Clinical Practice and the Declaration of Helsinki in compliance with all relevant national and international regulations. The study was approved by the METC of the UMC Amsterdam (METC) medical research ethics committee (MREC) and granted under code METC 2020_223 following national guidelines. The local ethics committee reviewed and approved the study and it was registered at the national body responsible for supervising clinical trials under code NL75272.018.20.

Medication was aseptically prepared at the pharmacy department. All analyses were performed within the Laboratory of Experimental Immunology within the Amsterdam University Medical Center. All work with SARS-CoV-2 was performed in a biosafety laboratory level 3 facility at our institution.

Volunteers were recruited from the University of Amsterdam. All participants provided written informed consent before any study procedures were performed.

### Participants.

Based on previous *in vitro* data, we expected to observe a reduction of 50% SARS-CoV-2 virus binding between treatment and placebo samples, to achieve a power of >80% at 0.05 statistical significance 12 volunteers would be needed per SARS-CoV-2 variant. The recruitment period lasted from January 7, 2021 until December 14, 2021, during this period a total of 36 volunteers were assessed for eligibility. Volunteer inclusion was done longitudinally to investigate whether all conditions could be performed for each volunteer, an extra volunteer would be included if this was not the case. Both genders were eligible to participate. Other inclusion criteria were age between 18 and 65 years, the ability to provide written informed consent, good physical health, which was defined as not suffering from any illness or disease obstructing general daily functioning, and sufficiently understanding Dutch in the opinion of the research physician taking informed consent. Exclusion criteria were positivity for SARS-CoV-2, as was tested during the study visit with a commercial SARS-CoV-2 antigen test (SARS-CoV-2 Antigen Rapid Test kit, JOYSBIO [Tianjin] Biotechnology Co. Ltd.), the presence of nasal-septum defects, usage of intranasal medication, frequent nosebleeds (defined as >1/month), a tympanic temperature exceeding 38.5°C during the clinical visit, anamnestic or physical evidence of respiratory infection in the 4 weeks before the clinical visits, a known allergy or intolerance to LMWH or heparin-related products, a medical history of heparin-induced thrombocytopenia (HIT), the presence of mental disorders that would interfere with adherence to study procedures or behavior making a volunteer unlikely to comply with study procedures in the opinion of the research physician present during the clinical visit. Vaccination status was self-reported with all volunteers receiving the enoxaparin treatment fully vaccinated according to national guidelines (1× COVID-19 vaccine Janssen, Janssen. 2× Comirnaty, BioNTech/Pfizer, or 2× Spikevax, Moderna mRNA vaccine). Additionally, 6 unvaccinated volunteers and 1 partially vaccinated volunteer were included for analysis of SARS-CoV-2 binding. However, these 7 volunteers were excluded from the enoxaparin block analysis due to insufficient treatment application. The inclusion of volunteers and processing of volunteer material is displayed in detail in Fig. 1.

### Enoxaparin administration.

Enoxaparin at a dose of 4500 IU and placebo (saline NaCl 0.9%) was administered in the nasal cavity using a nebulizer (MAD Nasal Intranasal Mucosal Atomization Device). We selected LMWH enoxaparin (Clexane Forte, 150 mg/mL) because we previously showed that it effectively blocked SARS-CoV-2 infection *in vitro* (28). Nebulization efficiency was investigated with a green tracer dye. Enoxaparin concentrations were not measured because this was technically challenging, but the same enoxaparin concentration was applied to every volunteer. Volunteers first received 370 μL of saline solution (NaCl 0.9%) in the left nasal cavity using a nebulizer (MAD Nasal Intranasal Mucosal Atomization Device). A total of 300 μL of fluid was administered, as 70 μL remained within the dead space of the nebulizer. Then, 100 μL was nebulized at 10-minute intervals to facilitate absorption by the nasal mucosa and epithelial cells were removed using a nasal brush (CytoSoft brush). Subsequently, 370 μL of LMWH enoxaparin (Clexane Forte, 150 mg/mL) was administered in the right nasal cavity identically as for the left nostril described above. After 30 min, cells were removed using a similar procedure. Participants were observed for the occurrence of adverse events during the study procedures. At 24 h after treatment follow-up was performed via a telephone call. Brushes containing nasal epithelium were stored in a 1.5 mL sterile microcentrifuge tube containing 0.5 mL of Iscove's modified Dulbecco's medium (IMDM), enriched with 10% fetal calf serum (FCS), 1% penicillin/streptomycin, 1% l-glutamine and 20 μg/mL gentamycin (Thermo Fischer Scientific, USA) to maintain the vitality of obtained cells.

### Nasal cell characterization.

Cell population characterization from the nasal tissue was performed by flow cytometric analyses of intracellular and cell surface expression markers. The gating strategy for FACS analysis is shown in Fig. 3. Single cells were further gated from the living population. Isolated cells were washed once with PBS and stained for Pan-Cytokeratin (panC) as well as CD45 to distinguish between epithelial or lymphoid origin. Additionally, cells were analyzed for the expression of ACE-2 and heparan sulfates (extracellular only) and other epithelial markers (EpCAM and Mucin 5b [Muc-5b]) as well as lymphoid markers CD3, CD11b, and CD11c. Some samples for flow cytometry, but not virus attachment analysis, were contaminated with erythrocytes and were excluded. Flow cytometry analysis was performed on a BD FACS Canto II (BD Biosciences) and data were analyzed using FlowJo v10.8.1 (Software by Treestar).

### VeroE6 culture.

VeroE6 cells (ATCC CRL-1586) were cultured in CO_2_ independent medium (Gibco Life Technologies, Gaithersburg, Md.) supplemented with 10% fetal calf serum (FCS), l-glutamine, and penicillin/streptomycin (10 μg/mL). The culture was maintained at 37°C without CO_2_. The VeroE6-ACE2-TMPRSS2 cell line (NISBC, 101003) expresses high levels of ACE2 and TMPRSS2 (66). These cells were maintained in Dulbecco’s modified Eagle’s high glucose medium supplemented with 10% FCS, 1% l-glutamine, and 1% penicillin/streptomycin. All reagents were obtained from Sigma-Aldrich, MO, USA) at 37°C with a saturation of 5% CO_2_.

### Viruses.

SARS-CoV-2 pseudovirus consists of a single round virus with an HIV-1 backbone (pNL4-3.Luc.R-S-) containing mutations in the capsid protein (as described in reference (67)) and a firefly luciferase gene in the nef open reading frame as well as pSARS-CoV-2 expressing SARS-CoV-2 S protein (GenBank accession no. MN908947.3) (68). The wild-type (WT) authentic SARS-CoV-2 virus hCoV-19/WT, was obtained from Maria R. Capobianchi through BEI Resources, NIAID, NIH: SARS-related coronavirus 2, isolate Italy-INMI1, NR-52284, originally isolated January 2020 in Rome, Italy. SARS-CoV-2 authentic virus stocks from primary isolates were produced in VeroE6 cells. Cytopathic effect (CPE) formation was closely monitored and after 48 h the virus supernatant was harvested. Viral titers were determined by 50% tissue culture infectious dose (TCID_50_) on VeroE6 cells. In short, VeroE6 cells were seeded in a 96-well plate at a cell density of 10,000 cells. The following day the cells were inoculated with a 5-fold serial dilution of SARS-CoV-2 isolate in quadruplicate. Cell cytotoxicity was measured using an MTT assay 48 h after infection. Loss of MTT staining as determined by a spectrometer (optical density at 580 nm [OD_580_]) was indicative of SARS-CoV-2-induced CPE. Viral titer was determined as TCID_50_/mL and calculated based on the method first proposed by Reed and Muench (69). SARS-CoV-2 WT virus (BEI Resources, Manassas, VA, USA; Center for Aids Reagents (CFAR)/National Institute for Biological Standards and Control (NIBSC); Nr-52281) was used for the 3D epithelial cell model of l human bronchial epithelial (NHBE) cultures and propagated according to the manufacturer’s instructions. Clinical specimens for SARS-CoV-2 VoC Delta (B.1.617.2) and Omicron (BA.5) were isolated from COVID-19-positive swabs (Ethics statement, ECS1166/2020) and cultured as previously described (70).

### *Ex vivo* SARS-CoV-2 binding to nasal epithelial cells.

After isolation from the nasal cavity, epithelial cells were subjected to SARS-CoV-2 pseudovirus and authentic SARS-CoV-2 virus. SARS-CoV-2 pseudovirus binding was performed with 100,000 cells per condition, while binding with the authentic virus was performed with 50,000 cells per condition. Cells were seeded in a 96-well round-bottom plate within 4 h of retrieval from the nasal cavity and incubated with either 95 ng/mL SARS-CoV-2 pseudovirus or 100 TCID_50_/mL of authentic virus (hCOV-19, WT) for 4 h at 4°C. To determine whether *in vivo* inhalation provided the most optimal block, a condition was included where viruses were incubated with 250 IU/mL of LMWH enoxaparin for 30 min at 37°C before being added to cells from volunteers. The cells were subsequently washed to remove any unbound virus. Cells incubated with SARS-CoV-2 pseudovirus were lysed after 4 h and binding and internalization were quantified by RETRO-TEK HIV-1 p24 enzyme-linked immunosorbent assay (ELISA) according to the manufacturer’s instructions (ZeptoMetrix Corporation). Cells incubated with SARS-CoV-2 isolate (hCoV-19/WT) were lysed with AVL buffer and RNA was isolated with the QIAamp Viral RNA Minikit (Qiagen) according to the manufacturer's protocol. cDNA was synthesized with the Moloney Murine Leukemia Virus (M-MLV) reverse transcriptase kit (Promega) and diluted 1 in 5 before further application. Virus quantification was performed using RT-PCR in the presence of SYBR green in a 7500 Fast Realtime PCR System (ABI). Primer sequences used for mRNA expression were for gene product: GAPDH, forward primer (CCATGTTCGTCATGGGTGTG), reverse primer (GGTGCTAA GCAGTTGGTGGTG). For gene product SARS-CoV-2 ORf1b, forward primer (TGGGGTTTTACAGGTAACCT), reverse primer (AACACGCTTAACAAAGCACTC) as described previously (71). The normalized amount of target mRNA was calculated from the Ct values obtained for both target and household mRNA with the equation Nt = 2Ct (GAPDH) − Ct(target).

### Enoxaparin application *in vitro*.

VeroE6 cells were seeded with 10,000 cells/well in a 96-well plate with CO_2_ independent medium. The following day, the authentic SARS-CoV-2 virus (10,500 TCID/mL) was incubated with different concentrations of enoxaparin for 30 min at 37C. Subsequently, the virus/enoxaparin mix was added to the VeroE6 cells. The binding of SARS-Cov-2 was determined after 4 h of incubation at 4°C whereas SARS-CoV-2 infection was measured after 24 h of incubation at 37°C. Alternatively, enoxaparin was applied directly to the cells for 30 min before exposure to authentic SARS-CoV-2. Binding and infection were performed as described above. SARS-CoV-2 levels were determined by RT-PCR as described above.

### 3D epithelial cell model in Caco-2 cells.

The human epithelial Caco-2 cell line (ATCC, HTB-37) was used for SARS-CoV-2 binding in addition to patient material. The cells were cultured on 5.0 μm microporous filters with an air-liquid interface to achieve a polarized monolayer in a static *in vitro* 3D epithelial model. The model was maintained in Dulbecco-modified Eagle medium (Gibco Life Technologies, Gaithersburg, Md.) containing 10% fetal calf serum (FCS), l-glutamine, and penicillin/streptomycin (10 μg/mL) and supplemented with MEM nonessential-amino-acids-solution (NEAA) (Gibco Life Technologies, Gaithersburg, Md.). Polarization was monitored by transepithelial electrical resistance (TEER) and full polarization was reached after 3 weeks in culture, as described previously (28). Caco-2 cells express ACE-2 as well as heparan sulfates. Upon full polarization, the cells were exposed to SARS-CoV-2 pseudovirus for 4 h at 4°C or authentic virus (hCOV-19/WT) for 24 h at 37°C before lysis.

### 3D epithelial cell model of normal human bronchial epithelial cells.

Normal human bronchial epithelial (NHBE, Lonza, catalog no. C-2540S) cells were cultured in air-liquid interphase (ALI) as described previously (41, 72). cells were seeded onto GrowDexT (UPM)-coated 0.33-cm^2^ porous (0.4-μm) polyester membrane inserts with a seeding density of 1 × 10^5^ cells per transwell (Costar, Corning, NY, USA). The cells were grown to near-confluence in submerged culture for 2 to 3 days in a specific epithelial cell growth medium according to the manufacturer’s instructions. Cultures were maintained in a humidified atmosphere with 5% CO2 at 37°C and then transferred to ALI culture. The epithelium was differentiated using an airway medium from Stemcell. Under these conditions, the cells develop stable tight junctions and are highly mucus-producing (72). The number of days in tissue development was designated relative to initiation of ALI culture, corresponding to day 0, and monitored by TEER. Enoxaparin spray was applied to the apical side of the fully polarized epithelia before the SARS-CoV-2 infection. The spray application corresponded to approximately 50 μL of liquid well dispersed over the tissue culture. The apical application was carefully performed to not mechanically disrupt the epithelial surface. TEER values were measured using an EVOM volt ohmmeter with STX-2 chopstick electrodes (World Precision Instruments, Stevenage, United Kingdom). Measurements on cells in ALI culture were taken immediately before the medium was exchanged. For measurements, 0.1 mL and 0.7 mL of medium were added to the apical and basolateral chambers, respectively.

SARS-CoV-2 was added at a concentration of a multiplicity of infection (MOI) of 0.1 for each authentic SARS-CoV-2 strain (WT, VoC Delta [B.1.617.2], and VoC Omicron [BA.5]). Infection was measured after 24 h incubation at 37°C. SARS-CoV-2 RNA (140 μL) was extracted using FavorPrep Viral RNA Minikit (FAVORGEN, Ping-Tung, Taiwan), according to the manufacturer’s instructions. Sequences specific to region N1 of the Nucleocapsid gene published on the CDC website (https://www.cdc.gov/coronavirus/2019-ncov/lab/rt-pcr-panel-primer-probes.html) were used. Luna Universal Probe One-Step RT-PCR kit (New England BioLabs, Ipswich, Mass) was used for target amplification, and runs were performed on the CFX96 real-time detection system (Bio-Rad). For absolute quantification using the standard curve method, SARS-CoV-2 RNA was obtained as a PCR standard control from the National Institute for Biological Standards and Control UK (Ridge, UK).

### Statistical analysis.

The power calculation of our sample size was based on our requirements for the primary endpoint. Based on this it was determined we would require sufficient cells of 12 volunteers to achieve >80% power with a certainty of 95%. Statistical analysis of obtained data was performed using GraphPad Prism 9 (GraphPad Software Inc.). Normal distribution of baseline data (age, sex, cell count per brush) was not assumed due to the distribution of samples and the small sample size. It was calculated for both cohorts using the Shapiro-Wilk test and found not to be normally distributed. A two-tailed Student's *t* test was used for paired observations, and Mann-Whitney tests were performed for unpaired observations. Differences between donors or cohorts were seen as paired, and differences between individual donors as unpaired. One-way analysis of variance (ANOVA), two-way ANOVA, and Mann-Whitney tests were performed for unpaired nonparametric observations. IC50 was calculated using a sigmoidal nonlinear regression. Statistical significance for our results was set *, *P* < 0.05; **, *P* < 0.01; ***, *P* < 0.001; and ****, *P* < 0.0001.

## References

[1] Zhou P, Yang XL, Wang XG, Hu B, Zhang L, Zhang W, Si HR, Zhu Y, Li B, Huang CL, Chen HD, Chen J, Luo Y, Guo H, Jiang RD, Liu MQ, Chen Y, Shen XR, Wang X, Zheng XS, Zhao K, Chen QJ, Deng F, Liu LL, Yan B, Zhan FX, Wang YY, Xiao GF, Shi ZL. 2020. A pneumonia outbreak associated with a new coronavirus of probable bat origin. Nature 579:270–273. doi:10.1038/s41586-020-2012-7. 32015507 PMC7095418

[2] Shivalkar S, Pingali MS, Verma A, Singh A, Singh V, Paital B, Das D, Varadwaj PK, Samanta SK. 2021. Outbreak of COVID-19: a Detailed Overview and Its Consequences, p 23–45. *In* Asea AAA, Kaur P (ed), Coronavirus Therapeutics – Volume II: Clinical Management and Public Health. Springer International Publishing, Cham. 10.1007/978-3-030-85113-2_2 35137366

[3] Wu F, Zhao S, Yu B, Chen YM, Wang W, Song ZG, Hu Y, Tao ZW, Tian JH, Pei YY, Yuan ML, Zhang YL, Dai FH, Liu Y, Wang QM, Zheng JJ, Xu L, Holmes EC, Zhang YZ. 2020. A new coronavirus associated with human respiratory disease in China. Nature 579:265–269. doi:10.1038/s41586-020-2008-3. 32015508 PMC7094943

[4] Lu R, Zhao X, Li J, Niu P, Yang B, Wu H, Wang W, Song H, Huang B, Zhu N, Bi Y, Ma X, Zhan F, Wang L, Hu T, Zhou H, Hu Z, Zhou W, Zhao L, Chen J, Meng Y, Wang J, Lin Y, Yuan J, Xie Z, Ma J, Liu WJ, Wang D, Xu W, Holmes EC, Gao GF, Wu G, Chen W, Shi W, Tan W. 2020. Genomic characterisation and epidemiology of 2019 novel coronavirus: implications for virus origins and receptor binding. Lancet 395:565–574. doi:10.1016/S0140-6736(20)30251-8. 32007145 PMC7159086

[5] Guan WJ, Ni ZY, Hu Y, Liang WH, Ou CQ, He JX, Liu L, Shan H, Lei CL, Hui DSC, Du B, Li LJ, Zeng G, Yuen KY, Chen RC, Tang CL, Wang T, Chen PY, Xiang J, Li SY, Wang JL, Liang ZJ, Peng YX, Wei L, Liu Y, Hu YH, Peng P, Wang JM, Liu JY, Chen Z, Li G, Zheng ZJ, Qiu SQ, Luo J, Ye CJ, Zhu SY, Zhong NS, China Medical Treatment Expert Group for Covid-19. 2020. Clinical characteristics of coronavirus disease 2019 in China. N Engl J Med 382:1708–1720. doi:10.1056/NEJMoa2002032. 32109013 PMC7092819

[6] Cummings MJ, Baldwin MR, Abrams D, Jacobson SD, Meyer BJ, Balough EM, Aaron JG, Claassen J, Rabbani LE, Hastie J, Hochman BR, Salazar-Schicchi J, Yip NH, Brodie D, O'Donnell MR. 2020. Epidemiology, clinical course, and outcomes of critically ill adults with COVID-19 in New York City: a prospective cohort study. Lancet 395:1763–1770. doi:10.1016/S0140-6736(20)31189-2. 32442528 PMC7237188

[7] Unicef. COVID-19 vaccine market dashboard. https://www.unicef.org/supply/covid-19-vaccine-market-dashboard. Accessed 11 February 2021.

[8] Cohn BA, Cirillo PM, Murphy CC, Krigbaum NY, Wallace AW. 2022. SARS-CoV-2 vaccine protection and deaths among US veterans during 2021. Science 375:331–336. doi:10.1126/science.abm0620. 34735261 PMC9836205

[9] Chen X, Huang H, Ju J, Sun R, Zhang J. 2022. Impact of vaccination on the COVID-19 pandemic in U.S. states. Sci Rep 12:1554. doi:10.1038/s41598-022-05498-z. 35091640 PMC8799714

[10] Emary KRW, Golubchik T, Aley PK, Ariani CV, Angus B, Bibi S, Blane B, Bonsall D, Cicconi P, Charlton S, Clutterbuck EA, Collins AM, Cox T, Darton TC, Dold C, Douglas AD, Duncan CJA, Ewer KJ, Flaxman AL, Faust SN, Ferreira DM, Feng S, Finn A, Folegatti PM, Fuskova M, Galiza E, Goodman AL, Green CM, Green CA, Greenland M, Hallis B, Heath PT, Hay J, Hill HC, Jenkin D, Kerridge S, Lazarus R, Libri V, Lillie PJ, Ludden C, Marchevsky NG, Minassian AM, McGregor AC, Mujadidi YF, Phillips DJ, Plested E, Pollock KM, Robinson H, Smith A, Song R, Oxford COVID-19 Vaccine Trial Group. 2021. Efficacy of ChAdOx1 nCoV-19 (AZD1222) vaccine against SARS-CoV-2 variant of concern 202012/01 (B.1.1.7): an exploratory analysis of a randomised controlled trial. Lancet 397:1351–1362. doi:10.1016/S0140-6736(21)00628-0. 33798499 PMC8009612

[11] Sanders RW, de Jong MD. 2021. Pandemic moves and countermoves: vaccines and viral variants. Lancet 397:1326–1327. doi:10.1016/S0140-6736(21)00730-3. 33798497 PMC8009609

[12] Moore JP, Offit PA. 2021. SARS-CoV-2 vaccines and the growing threat of viral variants. JAMA 325:821–822. doi:10.1001/jama.2021.1114. 33507218

[13] Collie S, Champion J, Moultrie H, Bekker LG, Gray G. 2022. Effectiveness of BNT162b2 vaccine against Omicron variant in South Africa. N Engl J Med 386:494–496. doi:10.1056/NEJMc2119270. 34965358 PMC8757569

[14] Sun J, Zheng Q, Madhira V, Olex AL, Anzalone AJ, Vinson A, Singh JA, French E, Abraham AG, Mathew J, Safdar N, Agarwal G, Fitzgerald KC, Singh N, Topaloglu U, Chute CG, Mannon RB, Kirk GD, Patel RC, Safo S, Patch DA, Haendel MA, Islam JY, Akselrod H, Franceschini N, Chiang TP, Bhattacharyya S, Bramante C, Duong T, Chirischilles EA, National COVID Cohort Collaborative (N3C) Consortium. 2022. Association between immune dysfunction and COVID-19 breakthrough infection after SARS-CoV-2 vaccination in the US. JAMA Internal Medicine 182:153–162. doi:10.1001/jamainternmed.2021.7024. 34962505 PMC8715386

[15] Ehmsen S, Asmussen A, Jeppesen SS, Nilsson AC, Østerlev S, Vestergaard H, Justesen US, Johansen IS, Frederiksen H, Ditzel HJ. 2021. Antibody and T cell immune responses following mRNA COVID-19 vaccination in patients with cancer. Cancer Cell 39:1034–1036. doi:10.1016/j.ccell.2021.07.016. 34348121 PMC8313483

[16] Geisen UM, Berner DK, Tran F, Sümbül M, Vullriede L, Ciripoi M, Reid HM, Schaffarzyk A, Longardt AC, Franzenburg J, Hoff P, Schirmer JH, Zeuner R, Friedrichs A, Steinbach A, Knies C, Markewitz RD, Morrison PJ, Gerdes S, Schreiber S, Hoyer BF. 2021. Immunogenicity and safety of anti-SARS-CoV-2 mRNA vaccines in patients with chronic inflammatory conditions and immunosuppressive therapy in a monocentric cohort. Ann Rheum Dis 80:1306–1311. doi:10.1136/annrheumdis-2021-220272. 33762264 PMC8117443

[17] Jarvis MC. 2020. Aerosol transmission of SARS-CoV-2: physical principles and implications. Front Public Health 8:590041. doi:10.3389/fpubh.2020.590041. 33330334 PMC7719704

[18] Rutter H, Parker S, Stahl-Timmins W, Noakes C, Smyth A, Macbeth R, Fitzgerald S, Freeman ALJ. 2021. Visualising SARS-CoV-2 transmission routes and mitigations. BMJ 375:e065312. doi:10.1136/bmj-2021-065312. 34853080

[19] Johnson TJ, Nishida RT, Sonpar AP, Lin YJ, Watson KA, Smith SW, Conly JM, Evans DH, Olfert JS. 2022. Viral load of SARS-CoV-2 in droplets and bioaerosols directly captured during breathing, speaking and coughing. Sci Rep 12:3484. doi:10.1038/s41598-022-07301-5. 35241703 PMC8894466

[20] Madas BG, Füri P, Farkas Á, Nagy A, Czitrovszky A, Balásházy I, Schay GG, Horváth A. 2020. Deposition distribution of the new coronavirus (SARS-CoV-2) in the human airways upon exposure to cough-generated droplets and aerosol particles. Sci Rep 10:22430. doi:10.1038/s41598-020-79985-6. 33384436 PMC7775446

[21] Hoffmann M, Kleine-Weber H, Schroeder S, Krüger N, Herrler T, Erichsen S, Schiergens TS, Herrler G, Wu NH, Nitsche A, Müller MA, Drosten C, Pöhlmann S. 2020. SARS-CoV-2 cell entry depends on ACE2 and TMPRSS2 and is blocked by a clinically proven protease inhibitor. Cell 181:271–280.e8. doi:10.1016/j.cell.2020.02.052. 32142651 PMC7102627

[22] Yang J, Petitjean SJL, Koehler M, Zhang Q, Dumitru AC, Chen W, Derclaye S, Vincent SP, Soumillion P, Alsteens D. 2020. Molecular interaction and inhibition of SARS-CoV-2 binding to the ACE2 receptor. Nat Commun 11:4541. doi:10.1038/s41467-020-18319-6. 32917884 PMC7486399

[23] Lan J, Ge J, Yu J, Shan S, Zhou H, Fan S, Zhang Q, Shi X, Wang Q, Zhang L, Wang X. 2020. Structure of the SARS-CoV-2 spike receptor-binding domain bound to the ACE2 receptor. Nature 581:215–220. doi:10.1038/s41586-020-2180-5. 32225176

[24] Lee IT, Nakayama T, Wu CT, Goltsev Y, Jiang S, Gall PA, Liao CK, Shih LC, Schürch CM, McIlwain DR, Chu P, Borchard NA, Zarabanda D, Dholakia SS, Yang A, Kim D, Chen H, Kanie T, Lin CD, Tsai MH, Phillips KM, Kim R, Overdevest JB, Tyler MA, Yan CH, Lin CF, Lin YT, Bau DT, Tsay GJ, Patel ZM, Tsou YA, Tzankov A, Matter MS, Tai CJ, Yeh TH, Hwang PH, Nolan GP, Nayak JV, Jackson PK. 2020. ACE2 localizes to the respiratory cilia and is not increased by ACE inhibitors or ARBs. Nat Commun 11:5453. doi:10.1038/s41467-020-19145-6. 33116139 PMC7595232

[25] Song X, Hu W, Yu H, Zhao L, Zhao Y, Zhao X, Xue HH, Zhao Y. 2020. Little to no expression of angiotensin-converting enzyme-2 on most human peripheral blood immune cells but highly expressed on tissue macrophages. Cytometry. doi:10.1002/cyto.a.24285. 33280254

[26] Clausen TM, Sandoval DR, Spliid CB, Pihl J, Perrett HR, Painter CD, Narayanan A, Majowicz SA, Kwong EM, McVicar RN, Thacker BE, Glass CA, Yang Z, Torres JL, Golden GJ, Bartels PL, Porell RN, Garretson AF, Laubach L, Feldman J, Yin X, Pu Y, Hauser BM, Caradonna TM, Kellman BP, Martino C, Gordts P, Chanda SK, Schmidt AG, Godula K, Leibel SL, Jose J, Corbett KD, Ward AB, Carlin AF, Esko JD. 2020. SARS-CoV-2 infection depends on cellular heparan sulfate and ACE2. Cell 183:1043–1057.e15. doi:10.1016/j.cell.2020.09.033. 32970989 PMC7489987

[27] Zhang Q, Chen CZ, Swaroop M, Xu M, Wang L, Lee J, Wang AQ, Pradhan M, Hagen N, Chen L, Shen M, Luo Z, Xu X, Xu Y, Huang W, Zheng W, Ye Y. 2020. Heparan sulfate assists SARS-CoV-2 in cell entry and can be targeted by approved drugs in vitro. Cell Discov 6:80. doi:10.1038/s41421-020-00222-5. 33298900 PMC7610239

[28] Bermejo-Jambrina M, Eder J, Kaptein TM, van Hamme JL, Helgers LC, Vlaming KE, Brouwer PJM, van Nuenen AC, Spaargaren M, de Bree GJ, Nijmeijer BM, Kootstra NA, van Gils MJ, Sanders RW, Geijtenbeek TBH. 2021. Infection and transmission of SARS-CoV-2 depend on heparan sulfate proteoglycans. EMBO J 40:e106765. doi:10.15252/embj.2020106765. 34510494 PMC8521309

[29] Sarrazin S, Lamanna WC, Esko JD. 2011. Heparan sulfate proteoglycans. Cold Spring Harb Perspect Biol 3:a004952. doi:10.1101/cshperspect.a004952. 21690215 PMC3119907

[30] Hayashida K, Johnston DR, Goldberger O, Park PW. 2006. Syndecan-1 expression in epithelial cells is induced by transforming growth factor beta through a PKA-dependent pathway. J Biol Chem 281:24365–24374. doi:10.1074/jbc.M509320200. 16807246

[31] Haeger SM, Liu X, Han X, McNeil JB, Oshima K, McMurtry SA, Yang Y, Ouyang Y, Zhang F, Nozik-Grayck E, Zemans RL, Tuder RM, Bastarache JA, Linhardt RJ, Schmidt EP. 2018. Epithelial heparan sulfate contributes to alveolar barrier function and is shed during lung injury. Am J Respir Cell Mol Biol 59:363–374. doi:10.1165/rcmb.2017-0428OC. 29584451 PMC6189644

[32] Milho R, Frederico B, Efstathiou S, Stevenson PG. 2012. A heparan-dependent herpesvirus targets the olfactory neuroepithelium for host entry. PLoS Pathog 8:e1002986. doi:10.1371/journal.ppat.1002986. 23133384 PMC3486907

[33] Tanino Y, Chang MY, Wang X, Gill SE, Skerrett S, McGuire JK, Sato S, Nikaido T, Kojima T, Munakata M, Mongovin S, Parks WC, Martin TR, Wight TN, Frevert CW. 2012. Syndecan-4 regulates early neutrophil migration and pulmonary inflammation in response to lipopolysaccharide. Am J Respir Cell Mol Biol 47:196–202. doi:10.1165/rcmb.2011-0294OC. 22427536 PMC3423465

[34] Haeger SM, Yang Y, Schmidt EP. 2016. Heparan sulfate in the developing, healthy, and injured lung. Am J Respir Cell Mol Biol 55:5–11. doi:10.1165/rcmb.2016-0043TR. 26982577 PMC4942210

[35] Nijmeijer BM, Eder J, Langedijk CJM, Kaptein TM, Meeussen S, Zimmermann P, Ribeiro CMS, Geijtenbeek TBH. 2020. Syndecan 4 upregulation on activated Langerhans cells counteracts langerin restriction to facilitate hepatitis C virus transmission. Front Immunol 11:503. doi:10.3389/fimmu.2020.00503. 32292405 PMC7118926

[36] de Witte L, Bobardt M, Chatterji U, Degeest G, David G, Geijtenbeek TB, Gallay P. 2007. Syndecan-3 is a dendritic cell-specific attachment receptor for HIV-1. Proc Natl Acad Sci USA 104:19464–19469. doi:10.1073/pnas.0703747104. 18040049 PMC2148312

[37] Milewska A, Zarebski M, Nowak P, Stozek K, Potempa J, Pyrc K. 2014. Human coronavirus NL63 utilizes heparan sulfate proteoglycans for attachment to target cells. J Virol 88:13221–13230. doi:10.1128/JVI.02078-14. 25187545 PMC4249106

[38] Tandon R, Sharp JS, Zhang F, Pomin VH, Ashpole NM, Mitra D, McCandless MG, Jin W, Liu H, Sharma P, Linhardt RJ. 2021. Effective inhibition of SARS-CoV-2 entry by heparin and enoxaparin derivatives. J Virol 95:e01987-20. doi:10.1128/JVI.01987-20. 33173010 PMC7925120

[39] Holbrook A, Schulman S, Witt DM, Vandvik PO, Fish J, Kovacs MJ, Svensson PJ, Veenstra DL, Crowther M, Guyatt GH. 2012. Evidence-based management of anticoagulant therapy: antithrombotic Therapy and Prevention of Thrombosis, 9th ed: American College of Chest Physicians Evidence-Based Clinical Practice Guidelines. Chest 141:e152S–e184S. doi:10.1378/chest.11-2295. 22315259 PMC3278055

[40] Cundiff DK, Manyemba J, Pezzullo JC. 2006. Anticoagulants versus non-steroidal anti-inflammatories or placebo for treatment of venous thromboembolism. Cochrane Database Syst Rev. doi:10.1002/14651858.CD003746.pub2. PMC7389637 16437461

[41] Zaderer V, Hermann M, Lass-Flörl C, Posch W, Wilflingseder D. 2019. Turning the world upside-down in cellulose for improved culturing and imaging of respiratory challenges within a human 3D model. Cells 8:1292. doi:10.3390/cells8101292. 31640299 PMC6830077

[42] Karantza V. 2011. Keratins in health and cancer: more than mere epithelial cell markers. Oncogene 30:127–138. doi:10.1038/onc.2010.456. 20890307 PMC3155291

[43] Anderson FA, Jr, Zayaruzny M, Heit JA, Fidan D, Cohen AT. 2007. Estimated annual numbers of US acute-care hospital patients at risk for venous thromboembolism. Am J Hematol 82:777–782. doi:10.1002/ajh.20983. 17626254

[44] Horlander KT, Mannino DM, Leeper KV. 2003. Pulmonary embolism mortality in the United States, 1979–1998: an analysis using multiple-cause mortality data. Arch Intern Med 163:1711–1717. doi:10.1001/archinte.163.14.1711. 12885687

[45] Gouin-Thibault I, Pautas E, Siguret V. 2005. Safety profile of different low-molecular weight heparins used at therapeutic dose. Drug Saf 28:333–349. doi:10.2165/00002018-200528040-00005. 15783242

[46] Markart P, Nass R, Ruppert C, Hundack L, Wygrecka M, Korfei M, Boedeker RH, Staehler G, Kroll H, Scheuch G, Seeger W, Guenther A. 2010. Safety and tolerability of inhaled heparin in idiopathic pulmonary fibrosis. J Aerosol Med Pulm Drug Deliv 23:161–172. doi:10.1089/jamp.2009.0780. 20109123

[47] Shute JK, Puxeddu E, Calzetta L. 2018. Therapeutic use of heparin and derivatives beyond anticoagulation in patients with bronchial asthma or COPD. Curr Opin Pharmacol 40:39–45. doi:10.1016/j.coph.2018.01.006. 29455115

[48] EMA. 2016. Inhixa: EPAR - product information. European Medicines Agency, EMA. https://www.ema.europa.eu/en/medicines/human/EPAR/inhixa. Accessed 9 July 2022.

[49] Hamming I, Timens W, Bulthuis ML, Lely AT, Navis G, van Goor H. 2004. Tissue distribution of ACE2 protein, the functional receptor for SARS coronavirus. A first step in understanding SARS pathogenesis. J Pathol 203:631–637. doi:10.1002/path.1570. 15141377 PMC7167720

[50] Wang C, Wang S, Chen Y, Zhao J, Han S, Zhao G, Kang J, Liu Y, Wang L, Wang X, Xu Y, Wang S, Huang Y, Wang J, Zhao J. 2021. Membrane nanoparticles derived from ACE2-rich cells block SARS-CoV-2 infection. ACS Nano 15:6340–6351. doi:10.1021/acsnano.0c06836. 33734675

[51] El-Shennawy L, Hoffmann AD, Dashzeveg NK, McAndrews KM, Mehl PJ, Cornish D, Yu Z, Tokars VL, Nicolaescu V, Tomatsidou A, Mao C, Felicelli CJ, Tsai C-F, Ostiguin C, Jia Y, Li L, Furlong K, Wysocki J, Luo X, Ruivo CF, Batlle D, Hope TJ, Shen Y, Chae YK, Zhang H, Lebleu VS, Shi T, Swaminathan S, Luo Y, Missiakas D, Randall GC, Demonbreun AR, Ison MG, Kalluri R, Fang D, Liu H. 2022. Circulating ACE2-expressing extracellular vesicles block broad strains of SARS-CoV-2. Nat Commun 13:405. doi:10.1038/s41467-021-27893-2. 35058437 PMC8776790

[52] Rajpoot S, Ohishi T, Kumar A, Pan Q, Banerjee S, Zhang KYJ, Baig MS. 2021. A Novel therapeutic peptide blocks SARS-CoV-2 spike protein binding with host cell ACE2 receptor. Drugs R D 21:273–283. doi:10.1007/s40268-021-00357-0. 34324175 PMC8319882

[53] Killingley B, Mann AJ, Kalinova M, Boyers A, Goonawardane N, Zhou J, Lindsell K, Hare SS, Brown J, Frise R, Smith E, Hopkins C, Noulin N, Löndt B, Wilkinson T, Harden S, McShane H, Baillet M, Gilbert A, Jacobs M, Charman C, Mande P, Nguyen-Van-Tam JS, Semple MG, Read RC, Ferguson NM, Openshaw PJ, Rapeport G, Barclay WS, Catchpole AP, Chiu C. 2022. Safety, tolerability and viral kinetics during SARS-CoV-2 human challenge in young adults. Nat Med 28:1031–1041. doi:10.1038/s41591-022-01780-9. 35361992

[54] Rapeport G, Smith E, Gilbert A, Catchpole A, McShane H, Chiu C. 2021. SARS-CoV-2 human challenge studies - establishing the model during an evolving pandemic. N Engl J Med 385:961–964. doi:10.1056/NEJMp2106970. 34289273

[55] Ahmed T, Gonzalez BJ, Danta I. 1999. Prevention of exercise-induced bronchoconstriction by inhaled low-molecular-weight heparin. Am J Respir Crit Care Med 160:576–581. doi:10.1164/ajrccm.160.2.9812076. 10430731

[56] Dixon B, Smith RJ, Campbell DJ, Moran JL, Doig GS, Rechnitzer T, Macisaac CM, Simpson N, Van Haren FMP, Ghosh AN, Gupta S, Broadfield EJC, Crozier TME, French C, Santamaria JD, CHARLI Study Group. 2021. Nebulised heparin for patients with or at risk of acute respiratory distress syndrome: a multicentre, randomised, double-blind, placebo-controlled phase 3 trial. Lancet Respiratory Medicine 9:360–372. doi:10.1016/S2213-2600(20)30470-7. 33493448 PMC7826120

[57] Tang N, Bai H, Chen X, Gong J, Li D, Sun Z. 2020. Anticoagulant treatment is associated with decreased mortality in severe coronavirus disease 2019 patients with coagulopathy. J Thromb Haemost 18:1094–1099. doi:10.1111/jth.14817. 32220112 PMC9906401

[58] Spyropoulos AC, Goldin M, Giannis D, Diab W, Wang J, Khanijo S, Mignatti A, Gianos E, Cohen M, Sharifova G, Lund JM, Tafur A, Lewis PA, Cohoon KP, Rahman H, Sison CP, Lesser ML, Ochani K, Agrawal N, Hsia J, Anderson VE, Bonaca M, Halperin JL, Weitz JI, Ohanesian L, Glater M, Ho C, Iakovou A, Ying D, Dastagir M, Convissar A, Aujla S, Mathew E, Thiyagarajan V, Lewis T, Gruberg L, Maccaro P, Kuziw D, Pandhi B, Surguladze G, Eapen AM, Pantea A, Suen P, Flynt J, Krzyzak M, Sharma K, Steadham A, McLean SL, Herring K, Maroney K, HEP-COVID Investigators., et al. 2021. Efficacy and safety of therapeutic-dose heparin vs standard prophylactic or intermediate-dose heparins for thromboprophylaxis in high-risk hospitalized patients with COVID-19. JAMA Internal Medicine 181:1612–1620. doi:10.1001/jamainternmed.2021.6203. 34617959 PMC8498934

[59] Paolisso P, Bergamaschi L, D'Angelo EC, Donati F, Giannella M, Tedeschi S, Pascale R, Bartoletti M, Tesini G, Biffi M, Cosmi B, Pizzi C, Viale P, Galie N. 2020. Preliminary experience with low molecular weight heparin strategy in COVID-19 patients. Front Pharmacol 11:1124. doi:10.3389/fphar.2020.01124. 32848743 PMC7424043

[60] Pereyra D, Heber S, Schrottmaier WC, Santol J, Pirabe A, Schmuckenschlager A, Kammerer K, Ammon D, Sorz T, Fritsch F, Hayden H, Pawelka E, Krüger P, Rumpf B, Traugott MT, Glaser P, Firbas C, Schörgenhofer C, Seitz T, Karolyi M, Pabinger I, Brostjan C, Starlinger P, Weiss G, Bellmann-Weiler R, Salzer HJF, Jilma B, Zoufaly A, Assinger A. 2021. Low-molecular-weight heparin use in coronavirus disease 2019 is associated with curtailed viral persistence: a retrospective multicentre observational study. Cardiovascular Res 117:2807–2820. doi:10.1093/cvr/cvab308. PMC8500043 34609480

[61] Trunfio M, Salvador E, Gaviraghi A, Audagnotto S, Marinaro L, Motta I, Casciaro R, Ghisetti V, Fava C, Bonora S, Di Perri G, Calcagno A, e-COVID Study Group. 2020. Early low-molecular-weight heparin administration is associated with shorter time to SARS-CoV-2 swab negativity. Antiviral Therapy 25:327–333. doi:10.3851/IMP3377. 33506810

[62] Shi C, Wang C, Wang H, Yang C, Cai F, Zeng F, Cheng F, Liu Y, Zhou T, Deng B, Vlodavsky I, Li JP, Zhang Y. 2020. The potential of low molecular weight heparin to mitigate cytokine storm in severe COVID-19 patients: a retrospective cohort study. Clin Transl Sci 13:1087–1095. doi:10.1111/cts.12880. 32881340 PMC7719364

[63] Cao Y, Wang J, Jian F, Xiao T, Song W, Yisimayi A, Huang W, Li Q, Wang P, An R, Wang J, Wang Y, Niu X, Yang S, Liang H, Sun H, Li T, Yu Y, Cui Q, Liu S, Yang X, Du S, Zhang Z, Hao X, Shao F, Jin R, Wang X, Xiao J, Wang Y, Xie XS. 2022. Omicron escapes the majority of existing SARS-CoV-2 neutralizing antibodies. Nature 602:657–663. doi:10.1038/d41586-021-03796-6. 35016194 PMC8866119

[64] Hudák A, Veres G, Letoha A, Szilák L, Letoha T. 2022. Syndecan-4 is a key facilitator of the SARS-CoV-2 Delta variant's superior transmission. Int J Mol Sci 23:796. doi:10.3390/ijms23020796. 35054983 PMC8775852

[65] Nie C, Sahoo AK, Netz RR, Herrmann A, Ballauff M, Haag R. 2022. Charge matters: mutations in Omicron variant favor binding to cells. Chembiochem 23:e202100681. doi:10.1002/cbic.202100681. 35020256 PMC9015620

[66] Matsuyama S, Nao N, Shirato K, Kawase M, Saito S, Takayama I, Nagata N, Sekizuka T, Katoh H, Kato F, Sakata M, Tahara M, Kutsuna S, Ohmagari N, Kuroda M, Suzuki T, Kageyama T, Takeda M. 2020. Enhanced isolation of SARS-CoV-2 by TMPRSS2-expressing cells. Proc Natl Acad Sci USA 117:7001–7003. doi:10.1073/pnas.2002589117. 32165541 PMC7132130

[67] Kootstra NA, Munk C, Tonnu N, Landau NR, Verma IM. 2003. Abrogation of postentry restriction of HIV-1-based lentiviral vector transduction in simian cells. Proc Natl Acad Sci USA 100:1298–1303. doi:10.1073/pnas.0337541100. 12547912 PMC298767

[68] Brouwer PJM, Caniels TG, van der Straten K, Snitselaar JL, Aldon Y, Bangaru S, Torres JL, Okba NMA, Claireaux M, Kerster G, Bentlage AEH, van Haaren MM, Guerra D, Burger JA, Schermer EE, Verheul KD, van der Velde N, van der Kooi A, van Schooten J, van Breemen MJ, Bijl TPL, Sliepen K, Aartse A, Derking R, Bontjer I, Kootstra NA, Wiersinga WJ, Vidarsson G, Haagmans BL, Ward AB, de Bree GJ, Sanders RW, van Gils MJ. 2020. Potent neutralizing antibodies from COVID-19 patients define multiple targets of vulnerability. Science 369:643–650. doi:10.1126/science.abc5902. 32540902 PMC7299281

[69] Reed LJ, Muench H. 1938. A simple method of estimating fifty percent endpoints. American J Epidemiology 27:493–497. doi:10.1093/oxfordjournals.aje.a118408.

[70] Tseng HF, Ackerson BK, Luo Y, Sy LS, Talarico CA, Tian Y, Bruxvoort KJ, Tubert JE, Florea A, Ku JH, Lee GS, Choi SK, Takhar HS, Aragones M, Qian L. 2022. Effectiveness of mRNA-1273 against SARS-CoV-2 Omicron and Delta variants. Nat Med 28:1063–1071. doi:10.1038/s41591-022-01753-y. 35189624 PMC9117141

[71] Chu DKW, Pan Y, Cheng SMS, Hui KPY, Krishnan P, Liu Y, Ng DYM, Wan CKC, Yang P, Wang Q, Peiris M, Poon LLM. 2020. Molecular diagnosis of a novel coronavirus (2019-nCoV) causing an outbreak of pneumonia. Clin Chem 66:549–555. doi:10.1093/clinchem/hvaa029. 32031583 PMC7108203

[72] Chandorkar P, Posch W, Zaderer V, Blatzer M, Steger M, Ammann CG, Binder U, Hermann M, Hörtnagl P, Lass-Flörl C, Wilflingseder D. 2017. Fast-track development of an in vitro 3D lung/immune cell model to study Aspergillus infections. Sci Rep 7:11644. doi:10.1038/s41598-017-11271-4. 28912507 PMC5599647

