# Supplementary figures and images for: Inhalation of Low Molecular Weight Heparins as Prophylaxis against SARS-CoV-2
Source: mBio. 2022 Nov 3;13(6):e02558-22. doi: 10.1128/mbio.02558-22 (PMC9765130; doi:10.1128/mbio.02558-22)

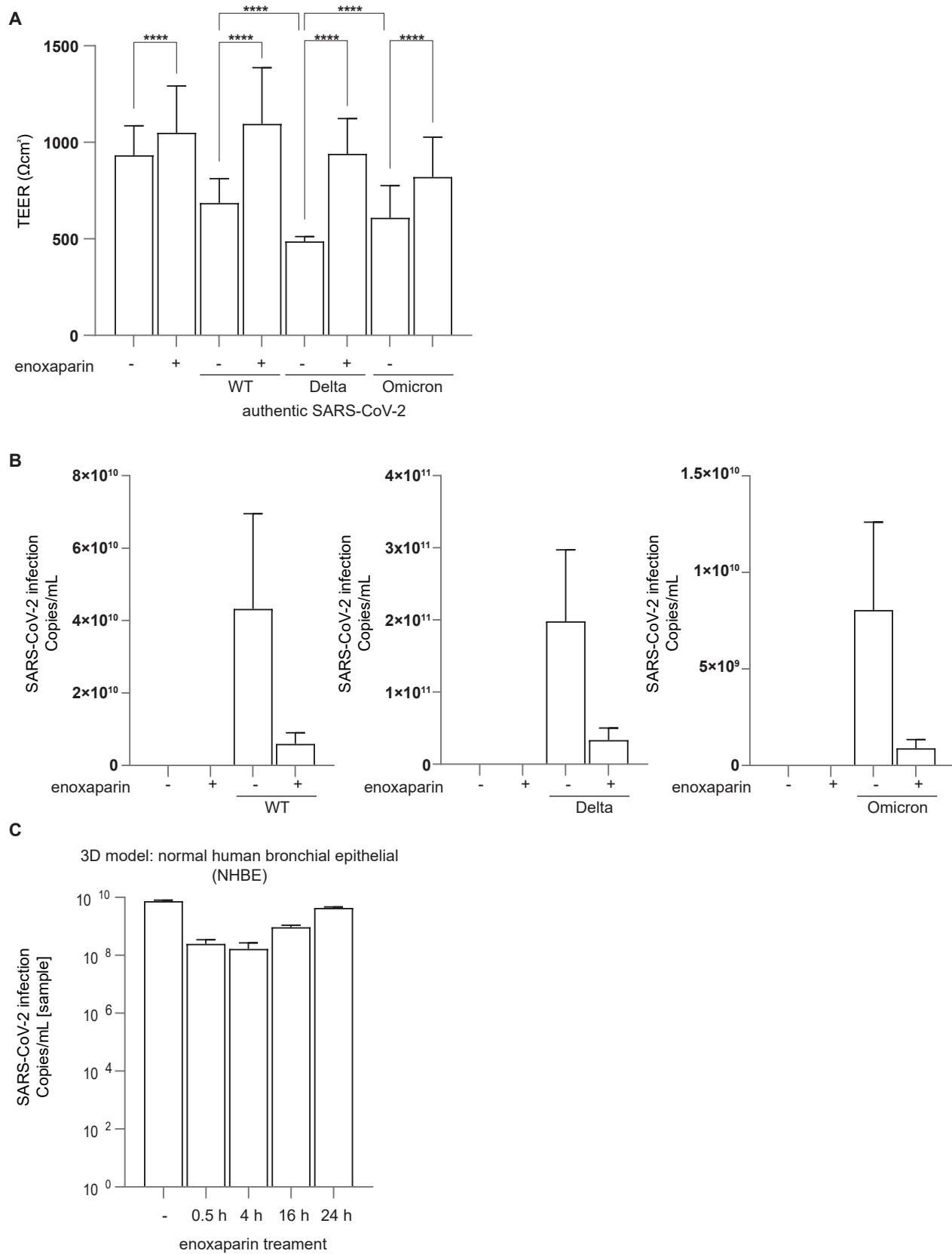

Supplemental Fig.1

Supplement: FIG S1 [file mbio.02558-22-s0001.pdf]

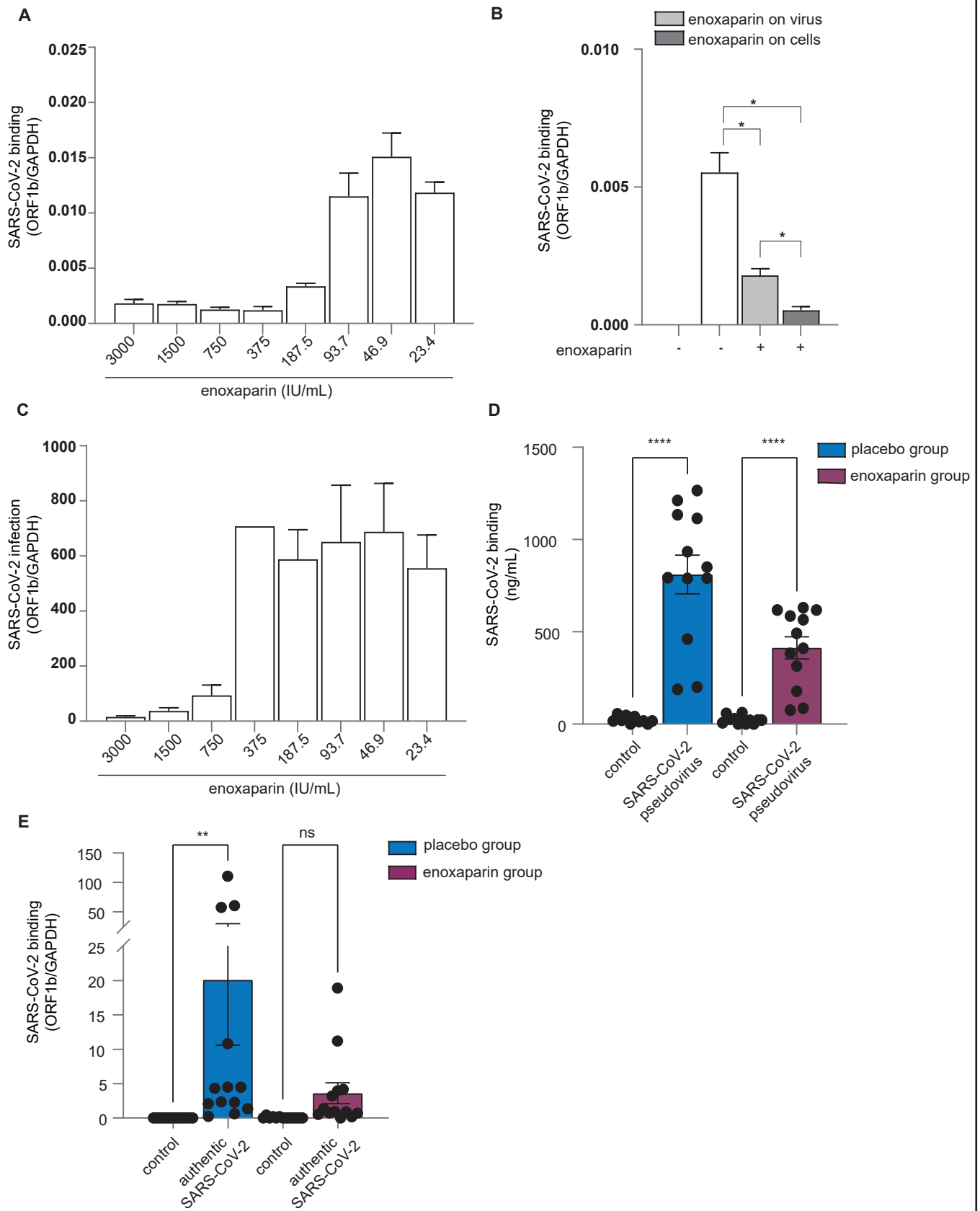

Supplemental Fig.2

Supplement: FIG S2 [file mbio.02558-22-s0002.pdf]
